# L-type voltage-gated calcium channel agonists mitigate hearing loss and modify ribbon synapse morphology in the zebrafish model of Usher syndrome type 1

**DOI:** 10.1242/dmm.043885

**Published:** 2020-11-27

**Authors:** Alaa Koleilat, Joseph A. Dugdale, Trace A. Christenson, Jeffrey L. Bellah, Aaron M. Lambert, Mark A. Masino, Stephen C. Ekker, Lisa A. Schimmenti

**Affiliations:** 1 College of Continuing and Professional Studies, University of Minnesota, Minneapolis, MN 55108, USA; 2 Mayo Clinic Graduate School of Biomedical Sciences, Clinical and Translational Science Track, Rochester, MN 55905, USA; 3 Center for Clinical and Translational Science, Mayo Clinic, Rochester, MN 55905, USA; 4 Department of Otorhinolaryngology, Mayo Clinic, Rochester, MN 55905, USA; 5 Mayo Clinic Microscopy and Cell Analysis Core, Rochester, MN 55905, USA; 6 Department of Genetics and Development, Columbia University, New York City, NY 10032, USA; 7 Department of Neuroscience, University of Minnesota, Minneapolis, MN 55455, USA; 8 Department of Molecular and Cellular Biology, Harvard University, Cambridge, MA 02138, USA; 9 Department of Biochemistry and Molecular Biology, Mayo Clinic, Rochester, MN 55905, USA; 10 Department of Pediatrics, University of Minnesota, Minneapolis, MN 55455, USA; 11 Department of Genetics, Cell Biology and Development, University of Minnesota, Minneapolis, MN 55455, USA; 12 Department of Ophthalmology and Visual Neuroscience, University of Minnesota, Minneapolis, MN 55454, USA; 13 Department of Clinical Genomics, Mayo Clinic, Rochester, MN 55905, USA

**Keywords:** Zebrafish, Hair cell, Ribbon synapse, *myo7aa*, Hearing loss

## Abstract

The *mariner* (*myo7aa^−/−^*) mutant is a zebrafish model for Usher syndrome type 1 (USH1). To further characterize hair cell synaptic elements in *myo7aa^−/−^* mutants, we focused on the ribbon synapse and evaluated ultrastructure, number and distribution of immunolabeled ribbons, and postsynaptic densities. By transmission electron microscopy, we determined that *myo7aa^−/−^* zebrafish have fewer glutamatergic vesicles tethered to ribbon synapses, yet maintain a comparable ribbon area. In *myo7aa^−/−^* hair cells, immunolocalization of Ctbp2 showed fewer ribbon-containing cells in total and an altered distribution of Ctbp2 puncta compared to wild-type hair cells. *myo7aa^−/−^* mutants have fewer postsynaptic densities – as assessed by MAGUK immunolabeling – compared to wild-type zebrafish. We quantified the circular swimming behavior of *myo7aa^−/−^* mutant fish and measured a greater turning angle (absolute smooth orientation). It has previously been shown that L-type voltage-gated calcium channels are necessary for ribbon localization and occurrence of postsynaptic density; thus, we hypothesized and observed that L-type voltage-gated calcium channel agonists change behavioral and synaptic phenotypes in *myo7aa^−/−^* mutants in a drug-specific manner. Our results indicate that treatment with L-type voltage-gated calcium channel agonists alter hair cell synaptic elements and improve behavioral phenotypes of *myo7aa^−/−^* mutants. Our data support that L-type voltage-gated calcium channel agonists induce morphological changes at the ribbon synapse – in both the number of tethered vesicles and regarding the distribution of Ctbp2 puncta – shift swimming behavior and improve acoustic startle response.

## Abstract (precis)

**Summary:** We quantified behavioral and synaptic morphology differences between wild-type zebrafish larvae and the *mariner* (*myo7aa^−/−^*) mutant, finding that these differences can be modified by L-type voltage-gated calcium channel agonists.

## INTRODUCTION

Up to 11% of deaf/hard of hearing children and 1 in 6000 people in the United States have Usher syndrome (USH) (Kimberling et al., 2010). USH is characterized by congenital onset of sensorineural deafness/hard of hearing and the later onset of retinitis pigmentosa, resulting in significant vision impairment (Usher, 1914). There are three types of USH, characterized by the degree of hearing loss, vestibular abnormalities and onset of vision loss (Davenport SLH, 1977).

Usher syndrome type I (USH1) is the most severe form of Usher syndrome, with profound bilateral congenital hearing loss and onset of retinitis pigmentosa within the first decade of life. Ten unique loci have been associated with USH1, with variants in *MYO7A* as the most common cause, accounting for 53-70% of affected individuals (Koenekoop et al., 1999). Additionally, pathogenic variants of *CDH23*, *PCDH15*, *USH1C* (also known as harmonin) and *USH1G* (also known as sans) are responsible for 19-35%, 11-19%, 6-7% and 7% of incidences, respectively (see the Hereditary Hearing Loss Homepage). Each gene encodes structural and motor proteins important for mechanotransduction in the inner ear hair cells (Beurg et al., 2009; Grati and Kachar, 2011; Grillet et al., 2009a; Kazmierczak et al., 2007; Marcotti, 2012; Pepermans and Petit, 2015; Siemens et al., 2004).

In 1995, Gibson et al. identified the first USH locus in the *shaker-1* (*sh1*) mouse (Gibson et al., 1995). The *sh1* mouse presented with hearing loss, head tossing and circling behaviors due to vestibular dysfunction, and upon examination of inner ear hair cells was found to have disorganized stereocilia. Through positional cloning techniques, homozygous mutations at the *sh1* locus were identified in *MYO7A*. Two years later, Weil et al. identified that USH1B in humans was also due to pathogenic variants of *MYO7A* (Weil et al., 1997). In 2000, Ernest et al. described a zebrafish model of USH1B caused by a premature stop codon in *myo7aa*, the *mariner* mutant, in which the phenotype of the homozygous recessive larval fish consisted of a circular swimming pattern, defective balance, morphological and functional defects of the inner ear hair cells and, most notably, the lack of a startle response (Ernest et al., 2000).

**Fig. 1.**
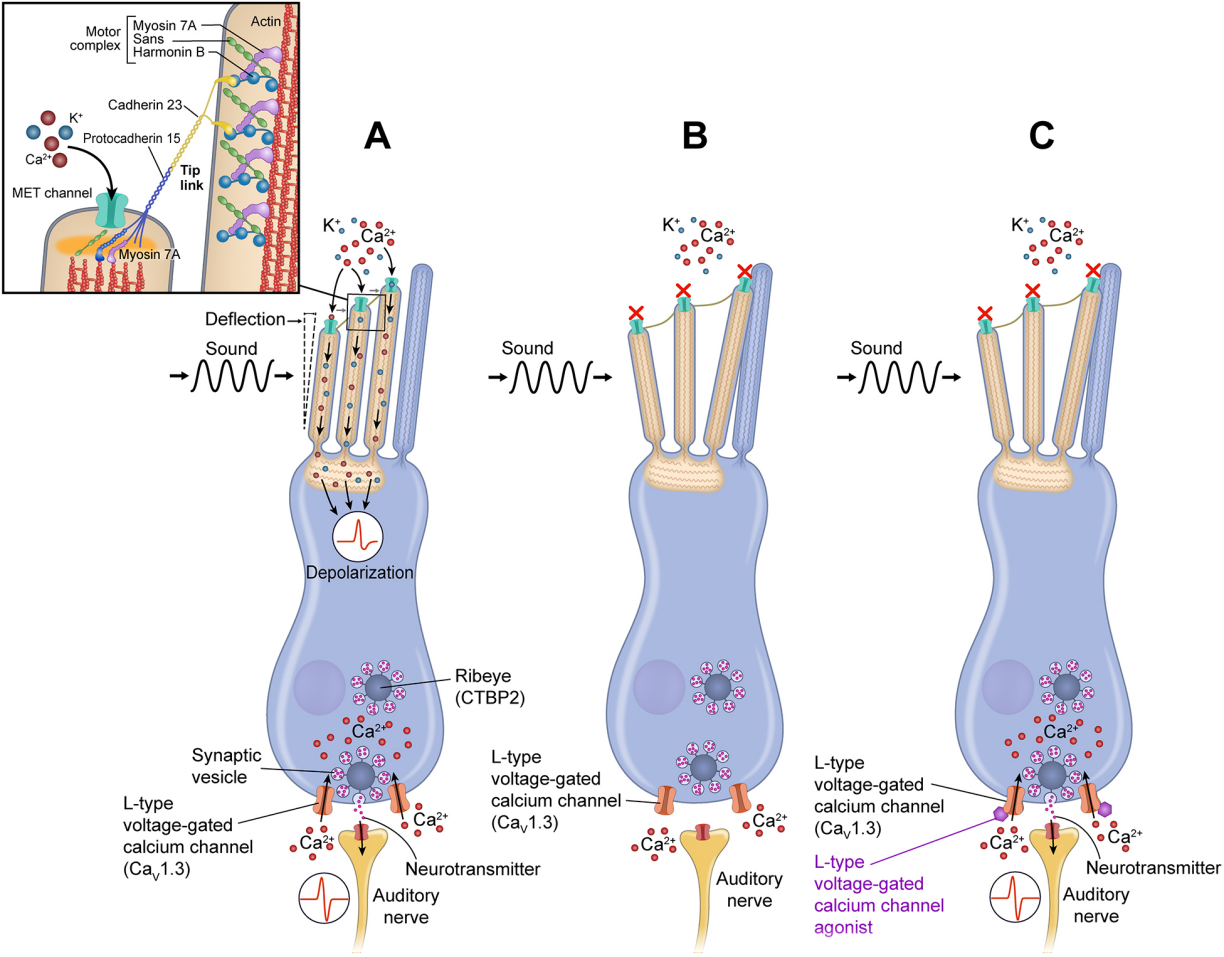
**L-type voltage-gated calcium channel agonists restore function in hair cells.** (A) In a normal hair cell, sound causes stereocilia to deflect towards the tallest stereocilium and induces the mechanotransduction channels (METs) at the top of the stereocilia to open in response, allowing cations such as calcium (Ca^2+^ ) and potassium (K^+^) to flow into the cell. This causes a change in membrane potential, which leads to the opening of L-type voltage-gated calcium channels at the basolateral sides of the cell. Calcium enters the cell and increases intracellular calcium concentrations, thereby mediating neurotransmitter release from synaptic vesicles within the ribbon synapse into the synaptic cleft, thus, stimulating afferent neurons. (B) In cells that lack MYO7A, correct MET channel gating does not occur. Therefore, the appropriate membrane potential is not reached to allow L-type voltage-gated calcium channels to open, and there is insufficient synaptic transmission to the auditory nerve to create meaningful interactions. (C) We hypothesize that, by augmenting the downstream signal in *myo7aa^−/−^* mutant hair cells, a new functional response to sound can be reconstituted when the sensitivity of the calcium channel is increased through treatment with L-type voltage-gated calcium channel agonists.

*MYO7A* encodes an unconventional actin-binding motor protein that is important for development and function of the inner ear hair cells. It is specifically involved in upholding the structural integrity of the hair bundle, allowing for a mechanical stimulus to be converted into a chemical stimulus. The MYO7A protein is localized at the upper tip link density of stereocilia in sensory hair cells (Hasson et al., 1995). In zebrafish, Myo7a, Ush1c and Ush1g interact with one another to connect the tip link end to the actin cytoskeleton of the stereocilium (Ahmed et al., 2006; Caberlotto et al., 2011; Grati and Kachar, 2011; Grillet et al., 2009b; Siemens et al., 2004). Myo7a is involved in maintaining the tension of the tip-link structure upon positive hair cell deflection. When sound is administered, the stereocilia of hair cells are deflected towards the tallest stereocilium allowing for the mechanoelectrical transduction channel (MET) located at the apical region of the stereocilia to open (Fig. 1A). The opening of the MET channel causes positively charged cations, such as potassium and calcium, to flow into the cell and affect depolarization.

Once depolarization occurs, L-type voltage-gated calcium channels (Ca_v_1.3) open, thereby increasing intracellular calcium concentrations (Brandt et al., 2005; Moser and Vogl, 2016; Sidi et al., 2004). Although calcium has many roles in sensory hair cells, entry of calcium through Ca_v_1.3 is necessary to mediate the release of synaptic vesicles tethered to the ribbon synapse structure (Fuchs, 2005; Moser et al., 2006; Nicolson, 2015; Wong et al., 2014). The ribbon synapse is primarily composed of a central protein, encoded by *CTBP2*, and surrounded by a halo of glutamatergic vesicles (Schmitz et al., 2000). When glutamate is released into the synaptic cleft, it binds onto postsynaptic cell receptors to stimulate cochlear afferents, thus, transducing the sound signal (Fig. 1A).

In the absence of *Myo7aa*, the MET channel is unable to gate properly in response to stereocilia deflections, therefore, cell depolarization is not reached and L-type voltage-gated calcium channels do not open, limiting synaptic transmission (Fig. 1B). In this study, we investigate the hypothesis that increasing intracellular calcium in the hair cell of a *myo7aa^−/−^* mutant mitigates hearing and swimming abnormalities and modifies synaptic elements. To address this hypothesis, we treated *myo7aa^−/−^* mutants with three different drugs, each of which induces some L-type voltage-gated calcium channel agonist activity. Drugs with L-type voltage-gated calcium channel activity have been previously used to treat other neurologic conditions, but not hearing loss (Hiramatsu et al., 1997; Kitano et al., 1994; Starkstein et al., 2016).

Here, we report that treatment of *myo7aa^−/−^* zebrafish with (±)-Bay K 8644, Nefiracetam and (R)-Baclofen can alter synaptic components and behavior observed in mutant zebrafish towards those of wild-type fish.

## RESULTS

### Hair cell ribbon synapse ultrastructure, Ctbp2 protein distribution and behavior of the *myo7aa*^−/−^ mutant differ from those of wild type

**Fig. 2.**
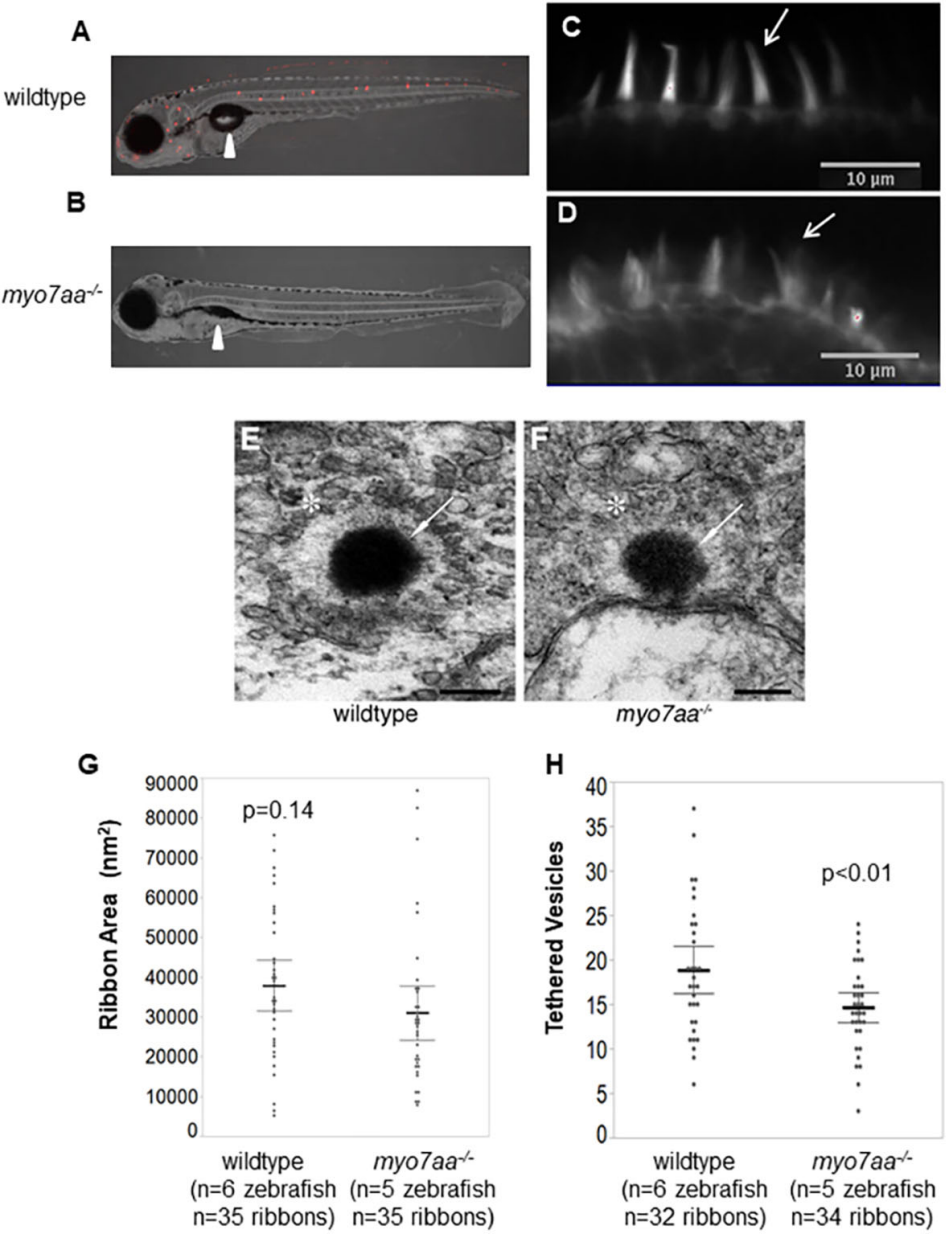
***myo7aa^−/−^* larvae have altered mechanotransduction activity, stereocilia structure and ribbon synapse structure.** (A,B) Representative light sheet fluorescence microscopy images showing the lateral view of 5 dpf wild-type (A) and *myo7aa^−/−^* (B) larvae after brief exposure to FM1-43. FM1-43 staining indicates functional mechanotransduction. Arrowheads point to the swim bladder; *myo7aa^−/−^* larvae do not have an inflated swim bladder. (C,D) Representative confocal microscopy images showing the stereocilia of the lateral crista of wild-type (C) and *myo7aa^−/−^* (D) larvae at 5 dpf, stained with Alexa-Fluor-488 tagged to phalloidin, a high-affinity filamentous actin probe. Arrows indicate the organized and smooth stereocilia in wild-type hair cells, and disorganized and splayed stereocilia in *myo7aa^−/−^* hair cells. Notice that not all stereocilia are splayed in *myo7aa^−/−^* hair cells. (E,F) Representative TEM images showing the ribbon synapse structures of 5 dpf wild-type (E) and *myo7aa^−/−^* (F) larvae. Arrows point to the ribbon density, stars indicate a halo of tethered vesicles in the inner ear hair cells. Scale bars: 200 nm. (G) Wild-type ribbon synapses have a comparable ribbon area to *myo7aa^−/−^* ribbon synapses (two-tailed *t*-test). (H) Wild-type ribbon synapses have an increased number of tethered vesicles compared to *myo7aa^−/−^* ribbon synapses (two-tailed *t*-test). The bold black lines represent the mean of the data set and error bars are 95% confidence intervals. Electron microscopy experiments were replicated four times for wild type and three times for *myo7aa^−/−^* mutants.

The *mariner* mutant (*myo7aa^−/−^* mutant) was first described in 2000 to have absent acoustic startle, balance abnormalities, a deflated swim bladder, and disorganized stereocilia (Ernest et al., 2000). It has previously been reported that the mechanotransduction channel does not uptake the vital dye FM 1-43 (Seiler and Nicolson, 1999). Transmission electron microscopy (TEM) imaging allowed ultrastructural evaluation of the ribbon synapse in inner ear hair cells of the lateral crista (Fig. 2E,F). The mean (±s.e.m.) of wild-type ribbon area is 37,866.7±3166.2 nm^2^ (*n*=35 ribbons; six zebrafish) and the mean of the *myo7aa^−/−^* mutants is 30,992.6±3347.2 nm^2^ (*n*=35 ribbons; five zebrafish); therefore, the ribbon area between *myo7aa^−/−^* mutants and wild-type larvae is comparable (Fig. 2G, *t*-test, *P*=0.14). The mean number of tethered vesicles for wild type is 19±1 vesicles (*n*=32 ribbons, six zebrafish), which is statistically different from the mean for the *myo7aa^−/−^* mutants 15±1 vesicles (*n*=34 ribbons, five zebrafish) (Fig. 2H, *t*-test, *P*<0.01).

**Fig. 3.**
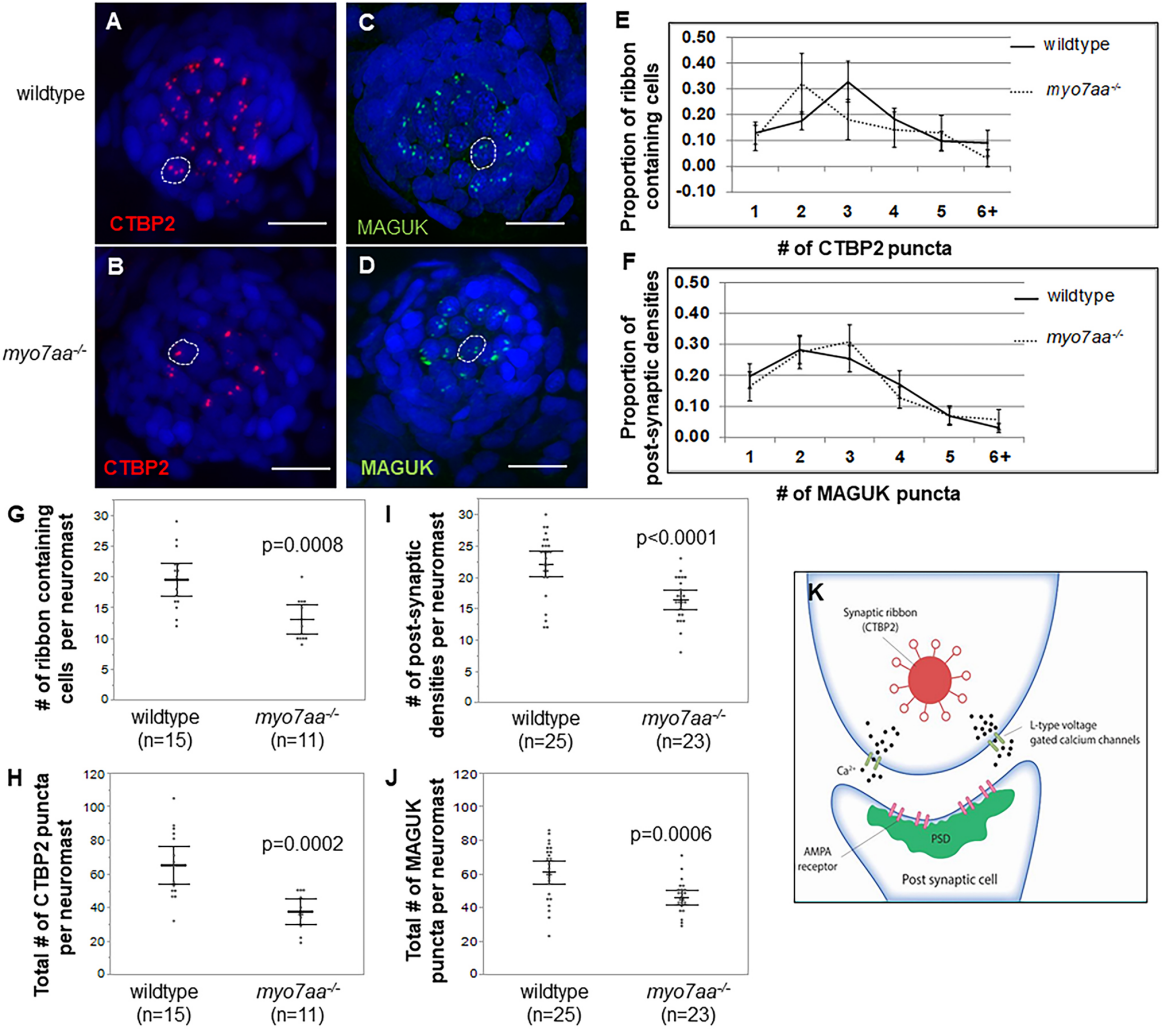
***myo7aa^−/−^* Ctbp2 and MAGUK synaptic elements are different from** **those of** **wild-type** **larvae.** (A,B) Representative maximum-intensity projection (*z*-stack top-down image) of MI1 neuromasts from 5 dpf wild-type larvae (A) and *myo7aa^−/−^* larvae (B). Nuclei were stained with DAPI (blue), Ctbp2 is shown in red. Dotted circles outline one hair cell. Scale bars: 10 µm. (C,D) Representative maximum-intensity projection (*z*-stack top-down image) of MI1 neuromasts from 5 dpf wild type (C) and *myo7aa^−/−^* (D). Nuclei were stained with DAPI (blue), MAGUK is shown in green. Dotted circles outline one cell. Scale bars: 10 µm. (E) Distribution of Ctbp2 puncta across a collection of ribbon-containing cells reveals that most 5 dpf wild-type ribbon-containing cells have two Ctbp2 puncta compared to three Ctbp2 puncta in *myo7aa^−/−^* hair cells. All error bars are 95% confidence intervals. (F) Distribution of MAGUK puncta across a collection of postsynaptic densities reveals that the proportion of postsynaptic densities is comparable between wild-type and *myo7aa^−/−^* larvae. (G,I) 5 dpf wild-type MI1 neuromasts have a greater number of ribbon-containing cells and greater total number of Ctbp2 puncta compared to *myo7aa^−/−^* MI1 neuromasts (two-tailed *t*-test). Bold black lines represent the mean of the data set and error bars are 95% confidence intervals. (H,J) 5 dpf wild-type MI1 neuromasts have a greater number of postsynaptic densities and a greater number of total MAGUK puncta compared to *myo7aa^−/−^* MI1 neuromasts (two-tailed *t*-test). Bold black lines represent the mean of the data set and error bars are 95% confidence intervals. (K) Depiction of the basal end of a hair cell innervated by the auditory nerve. Ctbp2 is alternatively spliced to produce Ribeye protein, the main component of the synaptic ribbon. Ribeye has a halo of tethered vesicles that form the synaptic ribbon (red). Members of the membrane associated guanylate kinase (MAGUK) superfamily are a part of the postsynaptic density (PSD), targeting and anchoring glutamate receptors to the synaptic terminals on the postsynaptic cell (green). Experiments were replicated five times for wild type for both Ctbp2 and MAGUK, and five times for *myo7aa^−/−^* mutants for Ctbp2 and two times for MAGUK.

To explore the ribbon synapse in *myo7aa^−/−^* mutants, we performed immunohistochemistry to assess differences in pre- and postsynaptic hair cell markers. Ctbp2 puncta were identified to determine the number of ribbon-containing cells per lateral line neuromast, specifically MI1 (Raible and Kruse, 2000), and the distribution of puncta per cell. Wild-type MI1 neuromasts have a mean of 20±5 ribbon-containing cells (*n*=15) and a mean of 65±20 total Ctbp2 puncta per neuromast (*n*=15). These two parameters are statistically different compared to the *myo7aa^−/−^* mutants that have a mean of 13±4 ribbon-containing cells (*n*=11) and a mean of 37±11 total Ctbp2 puncta per neuromast (*n*=11) (*t*-test, *P*=0.0008 and *P*=0.0002, respectively), indicating that there are fewer ribbon-containing cells and fewer Ctbp2 puncta per neuromast in the *myo7aa^−/−^* mutants (Fig. 3A,B,G,H). We also investigated the distribution of Ctbp2 puncta and identified that most hair cells from the *myo7aa^−/−^* mutant neuromasts had two puncta per cell and most wild-type hair cells had three puncta per cell (Fig. 3E). The proportion of hair cells with two Ctbp2 puncta and three Ctbp2 puncta was statistically significant between wild-type and *myo7aa^−/−^* mutants, and accompanied by no differences in the proportion of one, four, five and six+ Ctbp2 puncta between the two groups (Table S2).

We also explored any differences in the afferent postsynaptic structures in the afferent fiber terminal by using immunostaining against members of the postsynaptic hair cell marker membrane-associated guanylate kinase (MAGUK) superfamily (Anderson, 1996; Lv et al., 2016; Sheets et al., 2011), which target and anchor glutamate receptors to the synaptic terminal on the postsynaptic cell as part of the postsynaptic density (PSD) (Fig. 3K). Wild-type neuromasts had a mean of 22±5 postsynaptic densities (*n*=25) and a mean of 60±17 total MAGUK puncta per neuromast (*n*=25). These two parameters are statistically significant compared to those of *myo7aa^−/−^* mutants, which had a mean of 16±1 postsynaptic densities (*n*=23) and a mean of 46±2 total MAGUK puncta per neuromast (*n*=23) (*t*-test, *P*<0.0001 and *P*=0.0006, respectively). This indicated that there are fewer postsynaptic densities and fewer total MAGUK puncta per neuromast in the *myo7aa^−/−^* mutants (Fig. 3C,D,I,J). We also found no statistically significant difference in the distribution of MAGUK puncta between wild type and *myo7aa^−/−^* mutant (Fig. 3F; Table S2).

**Fig. 4.**
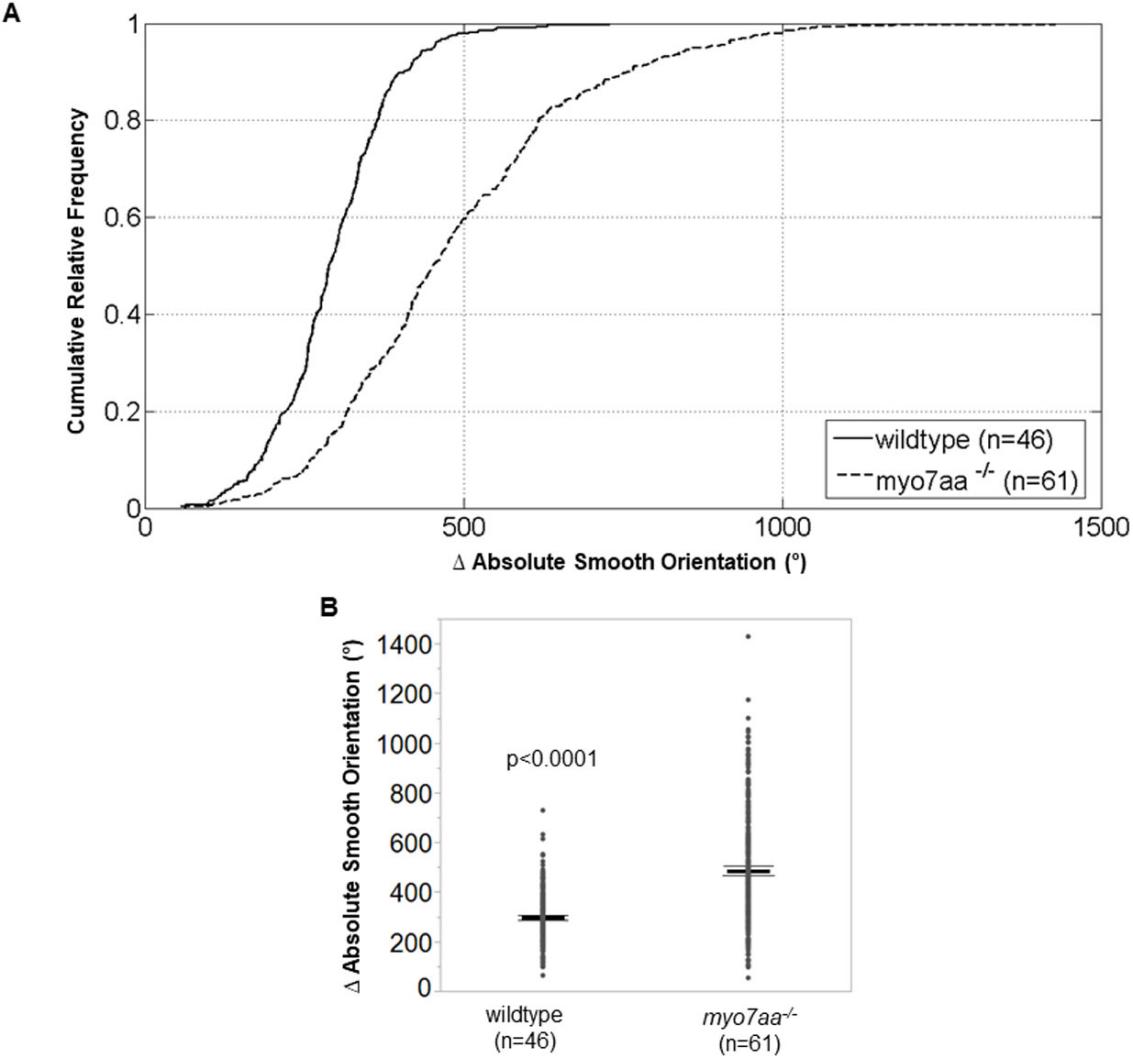
***myo7aa^−/−^* larvae exhibit larger turning angles.** Movement tracking of 5 dpf wild-type and *myo7aa^−/−^* larvae at 2.5-min intervals, with 5 ms electric stimuli (50 mV) administered every 20 s. Ctrax software was used for video processing and MATLAB 2012b for video analysis. (A,B) *myo7aa^−/−^* larvae have larger absolute smooth orientations (global change in body orientation) compared to wild-type larvae. Individual turning angles from a population of wild-type and *myo7aa^−/−^* larvae were used to construct the lines (two-tailed *t*-test). Bold black lines in B represent the mean of the data set and error bars are 95% confidence intervals. Experiments were replicated five times for both wild-type and *myo7aa^−/−^* mutants.

The abnormal circular and loop-like swimming behavior of the *myo7aa^−/−^* mutant is apparent when applying a tactile stimulus to the tail or through spontaneous swimming. We quantified swimming behavior by measuring the turning angle (absolute smooth orientation), defined as the sum of all turning angles a fish swims in one swimming episode as a function of time (Branson et al., 2009). The turning angle of wild-type fish was 295±5° (*n*=46) compared to that of the *myo7aa^−/−^* mutants which increased to 486±5° (*n*=61) (*t*-test; *P*<0.0001) (Fig. 4).

### L-type voltage-gated calcium channel agonists alter *myo7aa*^−/−^ ribbon synapse ultrastructure

**Fig. 5.**
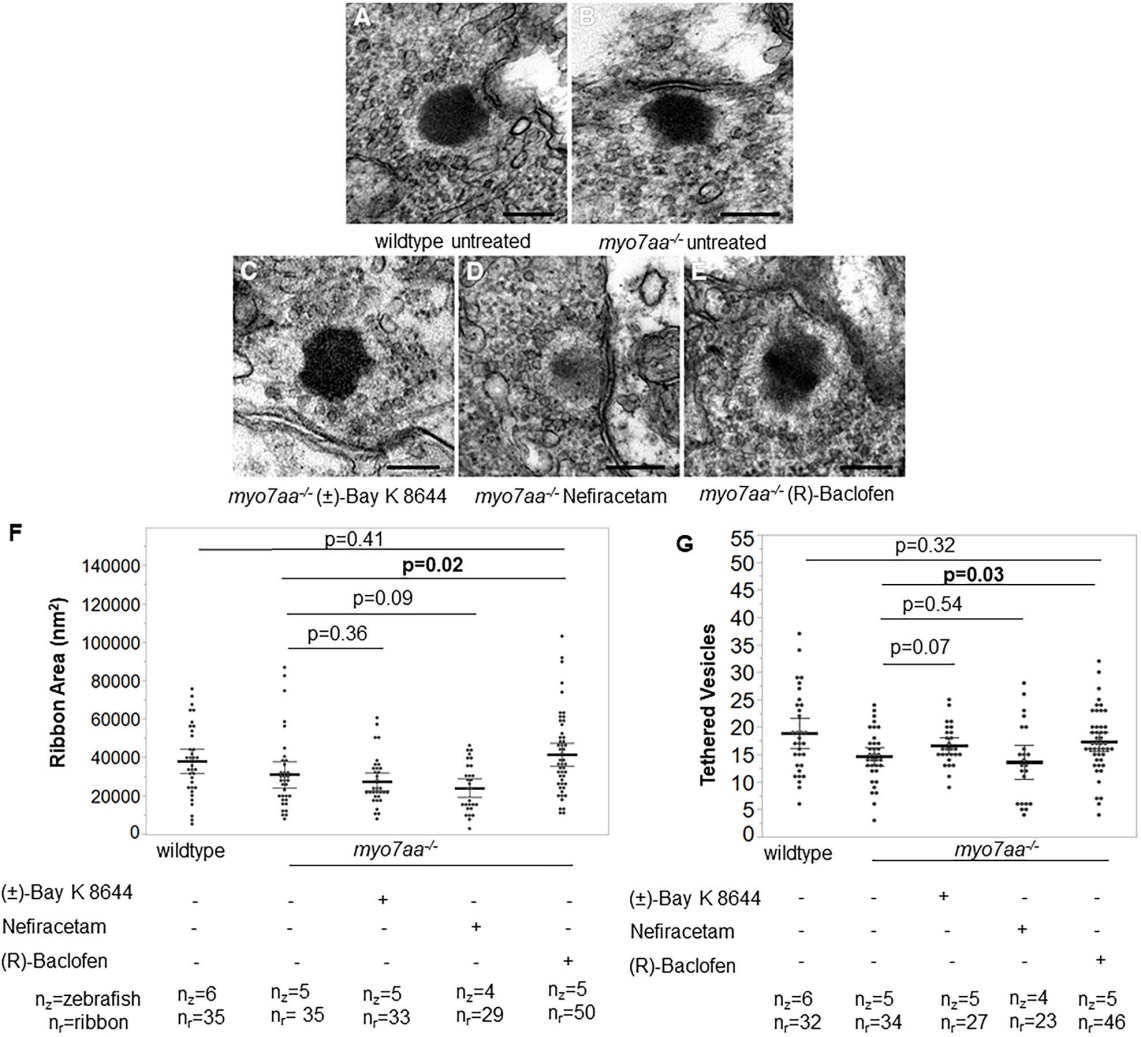
***myo7aa^−/−^* mutant ribbon synapse abnormalities** **are** **diminished** **after** **exposure to (R)-Baclofen.** (A-E) Representative TEM images showing ribbon synapses of untreated wild-type (A) and untreated *myo7aa^−/−^* mutant (B) larvae, as well as *myo7aa^−/−^* mutants treated with 5 µM (±)-Bay K 8644 (C), 250 µM Nefiracetam (D) or 125 µM (R)-Baclofen (E). All images were taken at 5 dpf. Scale bars: 1000 nm. (F) The ribbon area in untreated *myo7aa^−/−^* is smaller compared to that in *myo7aa^−/−^* treated with 125 µM (R)-Baclofen. Ribbon synapses of untreated wild-type larvae have a ribbon area comparable to that of *myo7aa^−/−^* mutants treated with 125 µM (R)-Baclofen (two-tailed *t*-test). (G) Ribbons of untreated *myo7aa^−/−^* mutants have fewer tethered vesicles compared to those of *myo7aa^−/−^* mutants treated with 125 µM (R)-Baclofen. Ribbons of untreated wild-type larvae have a number of tethered vesicles comparable to those of *myo7aa^−/−^* larvae treated with 125 µM (R)-Baclofen (two-tailed *t*-test). Bold black lines represent the mean of the data set and error bars are 95% confidence intervals. Experiments were replicated three times for *myo7aa^−/−^* mutants treated with each drug.

To explore whether L-type voltage-gated calcium channel agonists modify components of the hair cell ribbon synapse, we incubated 4 days post fertilization (dpf) *myo7aa^−/−^* mutant embryos overnight in 5 μM (±)-Bay K 8644, 250 μM Nefiracetam or 125 μM (R)-Baclofen, and fixed for TEM at 5 dpf. We observed that the mean area of the ribbon density for *myo7aa^−/−^* mutant fish incubated with either 5 μM (±)-Bay K 8644 or 250 μM Nefiracetam was, respectively, 27,274.97±2231.61 nm^2^ (*n*=35 ribbons, *n*=5 zebrafish) (*t*-test, *P*=0.36) or 24,024.14±2335.09 nm^2^ (*n*=29 ribbons, *n*=4 zebrafish) (*t*-test, *P*=0.09) and, thus, did not alter the ribbon density area. Additionally, neither drug altered the number of vesicles tethered to the ribbon density, with a mean of 17±1 (*n*=35 ribbons, *n*=5 zebrafish) (*t*-test, *P*=0.07) and a mean of 14±1 (*n*=29 ribbons, *n*=4 zebrafish) (*t*-test, *P*=0.54), respectively. Treatment with 125 μM (R)-Baclofen modified both parameters, with a mean area of 44,071.6±3008 nm^2^ (*n*=50 ribbons, *n*=5 zebrafish) (*t*-test, *P*=0.02) and 17±1 tethered vesicles (*n*=46 ribbons, *n*=5 zebrafish) (*t*-test, *P*=0.03) (Fig. 5). None of the three drugs showed any adverse effects on wild-type ribbon synapse ultrastructure (Fig. S1).

### L-type voltage-gated calcium channel agonists alter distribution of Ctbp2 puncta

**Fig. 6.**
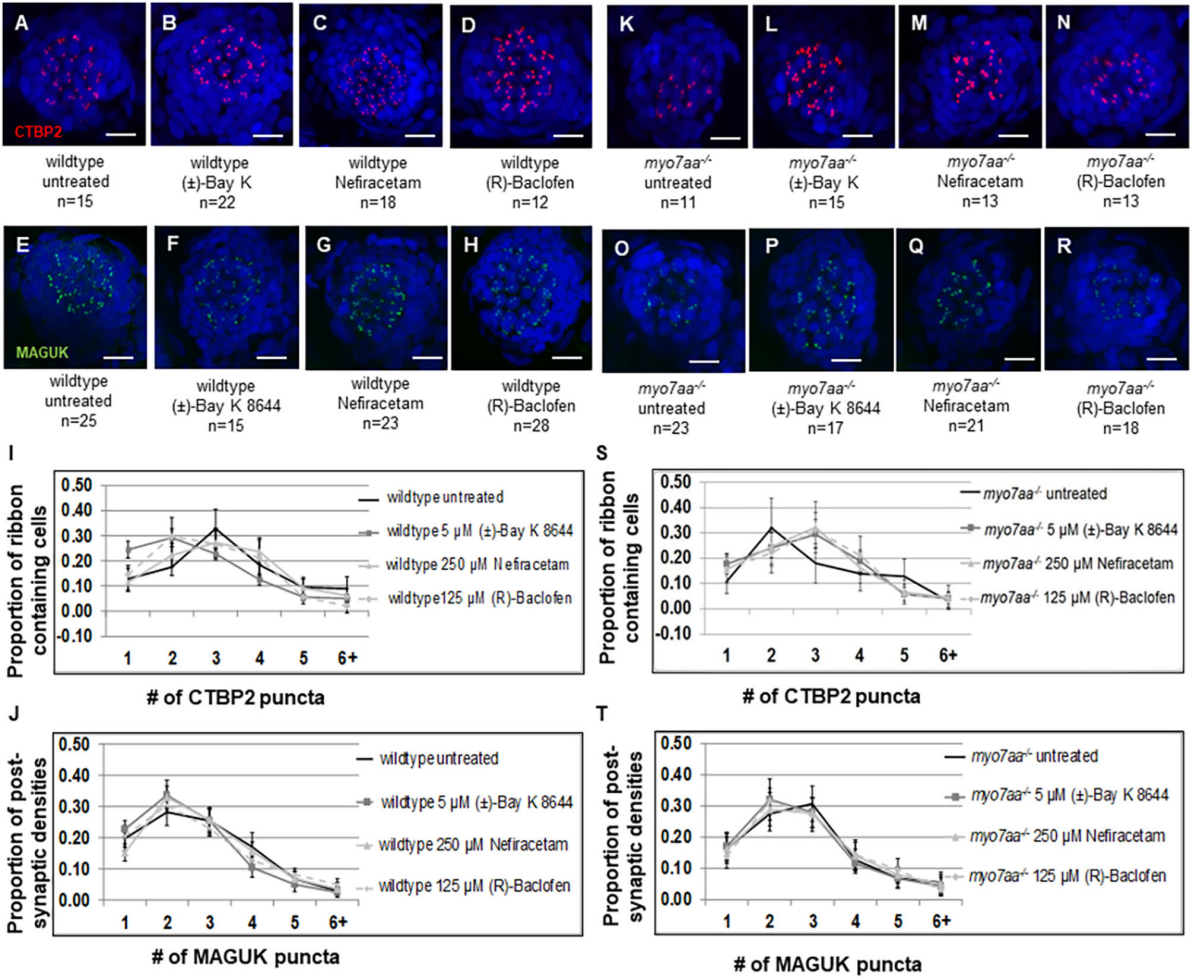
***myo7aa^−/−^* neuromasts** **exposed to L-type voltage-gated calcium channel agonists show a Ctbp2 distribution which more closely resembles that in wild-type larvae.** (A-D,K-N) Representative maximum-intensity projection (*z*-stack top-down image) of MI1 neuromasts from untreated and treated 5 dpf wild-type larvae (A-D) and untreated and treated *myo7aa^−/−^* larvae (K-N). Nuclei were labeled with DAPI (blue), staining for Ctbp2 is shown in red. Scale bars: 10 µm. (I) The majority of 5 dpf untreated wild-type ribbon-containing cells show three Ctbp2 puncta, with a statistical significance of wild-type larvae treated with 5 µM (±)-Bay K 8644 showing one, two, three or four puncta and those treated with 125 µM (R)-Baclofen showing two puncta. There was no statistically significant change upon incubation with 250 µM Nefiracetam. (S) The majority of 5 dpf untreated *myo7aa^−/−^* ribbon-containing cells have two Ctbp2 puncta. Upon incubation with 5 µM (±)-Bay K 8644, 250 µM Nefiracetam or 125 µM (R)-Baclofen the majority of hair cells have three Ctbp2 puncta, with a statistical significance of three puncta in all treatment groups. (E-H,O-R) Representative maximum intensity projection (*z*-stack top-down image) of MI1 neuromasts from 5 dpf wild-type larvae untreated and treated (E-H) and *myo7aa^−/−^* larvae untreated and treated (O-R). Nuclei were labeled with DAPI (blue), staining for MAGUK is shown in green. Scale bars: 10 µm. (J) There is no statistical significance in the proportion of one, two, three, four, five or six+ MAGUK puncta between untreated wild-type larvae and any of the treatment groups – except the occurrence of four puncta in response to treatment with 5 µM (±)-Bay K 8644. (T) There is no statistical significance in the proportion of one, two, three, four, five or six+ MAGUK puncta between untreated *myo7aa^−/−^* and any of the treatment groups. All error bars are 95% confidence intervals. Experiments for which Ctbp2 staining was carried out in wild-type animals were replicated twice for 5 µM (±)-Bay K 8644, three times for 250 µM Nefiracetam and twice for 125 µM (R)-Baclofen; MAGUK-labeling experiments were conducted twice for 5 µM (±)-Bay K 8644, three times for 250 µM Nefiracetam and three times for 125 µM (R)-Baclofen. Experiments for which Ctbp2 staining was carried out in *myo7aa^−/−^* mutants were replicated twice for 5 µM (±)-Bay K 8644, twice for 250 µM Nefiracetam and three times for 125 µM (R)-Baclofen; MAGUK-labeling experiments were conducted twice for 5 µM (±)-Bay K 8644, three times for 250 µM Nefiracetam and three times for 125 µM (R)-Baclofen.

We then explored the effect of L-type voltage-gated calcium channel agonists on Ctbp2 and MAGUK distribution. Wild-type larvae treated with 250 μM Nefiracetam did not show altered distribution of Ctbp2 puncta. However, incubation with 5 μM (±)-Bay K 8644 resulted in a statistically significant change, with one, two, three or four Ctbp2 puncta and four MAGUK puncta (Fig. 6A-C,F,I,J; Tables S3 and S4). There was a statistically significant difference at two Ctbp2 puncta upon incubation with 125 μM (R)-Baclofen (Fig. 6D,J; Table S3). Additionally, there was an increase in the total number of ribbon-containing cells per neuromast for wild-type fish incubated in 5 μM (±)-Bay K 8644, but no statistical difference in either parameter in response to 250 μM Nefiracetam or 125 μM (R)-Baclofen (Fig. S2A). We also observed an increase in the number of postsynaptic densities per neuromast for wild-type fish incubated in 5 μM (±)-Bay K 8644 or 125 μM (R)-Baclofen, but no change with 250 μM Nefiracetam (Fig. S3A). We also observed an increase in the total number of MAGUK puncta per neuromast for wild-type fish incubated in 125 μM (R)-Baclofen (Fig. S3A).

We identified that *myo7aa^−/−^* mutant larvae treated with 5 μM (±)-Bay K 8644, 250 μM Nefiracetam or 125 μM (R)-Baclofen showed altered distribution of Ctbp2 puncta. There was a statistically significant difference in the proportion of ribbon-containing cells with two Ctbp2 puncta in all treatment groups (Fig. 6K-N,S; Table S3). There was no statistically significant change in the distribution of MAGUK puncta in response to incubation with any of the three compounds (Fig. 6O-R,T; Table S4). Moreover, there was an increase in the total number of ribbon-containing cells per neuromast, total number of Ctbp2 puncta per neuromast, total number of postsynaptic densities per neuromast and total number of MAGUK puncta per neuromast for *myo7aa^−/−^* fish incubated in 5 μM (±)-Bay K 8644 (Figs S2B and S3B).

### L-type voltage-gated calcium channel agonists restore *myo7aa*^−/−^ swimming

**Fig. 7.**
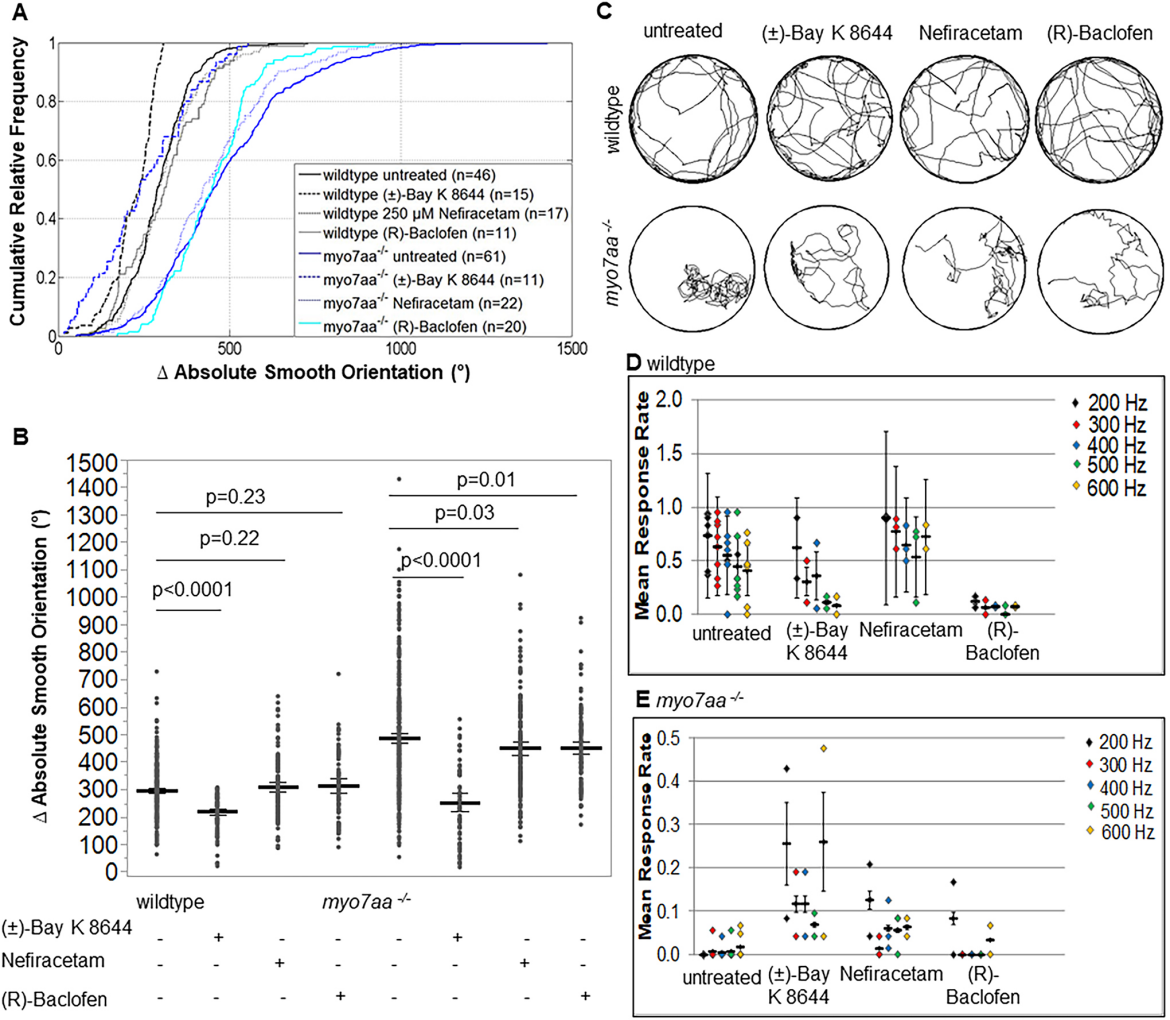
***myo7aa^−/−^* swimming behavior and acoustic startle response improved** **during treatment with** **L-type voltage-gated calcium channel agonists.** Movement tracking of 5 dpf wild-type and *myo7aa^−/−^* larvae over a 2.5-min interval with a 5 ms electric stimulus (50 mV) administered every 20 s. Ctrax software was used for video processing and MATLAB 2012b for video analysis. (A,B) 250 µM Nefiracetam and 125 µM (R)-Baclofen did not affect the absolute smooth orientation (global change in body orientation) of wild-type larvae; however, 5 µM (±)-Bay K 8644 resulted in a decreased absolute smooth orientation compared to that of untreated wild-type larvae. When *myo7aa^−/−^* larvae were individually treated with 5 µM (±)-Bay K 8644, 250 µM Nefiracetam or 125 µM (R)-Baclofen, either treatment decreased absolute smooth orientations, with the most robust response observed during incubation with 5 µM (±)-Bay K 8644. Individual turning angles from a population of wild-type and *myo7aa^−/−^* larvae were used to construct the lines (two-tailed *t*-test). Bold black lines represent the mean of the data sets and error bars are 95% confidence intervals. Experiments were conducted twice for each drug treatment. (C) Sample movement tracing of wild-type and *myo7aa^−/−^* larvae within individual treatment groups indicates that in the presence of 5 µM (±)-Bay K 8644, 250 µM Nefiracetam or 125 µM (R)-Baclofen induces changes to the swimming of *myo7aa^−/−^* larvae that include smoother trajectories with more zig-zag-like swimming and fewer circling episodes. Diameter of the wells was 20 mm. (D,E) Acoustic startle response was captured by administering three stimuli at each frequency per experiment. Videos were scored blindly and the mean number of responses per total stimuli (mean response rate) was determined. *myo7aa^−/−^* larvae showed little to no response at all frequencies. Upon incubation with 5 µM (±)-Bay K 8644 acoustic startle response in wild-type larvae decreased at all frequencies; however, *myo7aa^−/−^* larvae showed a significant increase, with a response rate of >20% at 200 and 600 Hz. Incubation in 250 µM Nefiracetam increased acoustic startle response in wild-type and *myo7aa^−/−^* larvae at all frequencies, although the increase in the *myo7aa^−/−^* larvae is modest. 125 µM (R)-Baclofen decreased acoustic startle response significantly in wild-type larvae and had little to no effect on acoustic startle in the *myo7aa^−/−^* larvae. Bold black lines represent the mean of the data set and error bars are the mean variance. Experiments were conducted eight times for wild-type and *myo7aa^−/−^* mutant controls, twice for wild-type and *myo7aa^−/−^* mutants incubated in 5 µM (±)-Bay K 8644 or 125 µM (R)-Baclofen, three times for wild type incubated in 250 µM Nefiracetam, and twice for *myo7aa^−/−^* larvae incubated in 250 µM Nefiracetam.

Wild-type fish incubated in 5 μM (±)-Bay K 8644 exhibit smaller turning angles with a mean of 218±6° (*n*=15 zebrafish) compared to untreated wild-type larvae with a mean of 295±5° (*n*=46 zebrafish) (*t*-test; *P*<0.0001) (Fig. 7A,B). Wild-type animals incubated with 250 μM Nefiracetam or 125 μM (R)-Baclofen have similar turning angles of 309±10° (*n*=17 zebrafish) and 313±13° (*n*=11) (*t*-test, *P*=0.22 and *P*=0.23, respectively), and swimming trajectories compared to untreated wild-type fish (Fig. 7A,B).

*myo7aa^−/−^* mutant fish incubated in 5 μM (±)-Bay K 8644, 250 μM Nefiracetam or 125 μM (R)-Baclofen showed improved swimming behavior, with decreased turning angles of 252±16° (*n*=11), 450±13° (*n*=22) and 451±11° (*n*=20) (*t*-test, *P*<0.0001, *P*=0.03 and *P*=0.01, respectively) compared to untreated controls (Fig. 7A,B). *myo7aa^−/−^* fish incubated with any of the three drugs displayed less circular swimming and loop-like swimming (Fig. 7C).

We conducted dose-response and time-response swimming behavior assessments for wild-type fish and *myo7aa*^−/−^ mutants treated with Nefiracetam or (R)-Baclofen (Figs S4-S7). We tested three doses of Nefiracetam, i.e. 85 μM, 125 μM and 250 μM. Turning angles for drug-treated wild-type fish did not differ from those of untreated fish except at 125 μM; and turning angles for drug-treated *myo7aa^−/−^* mutant fish did not differ from those of untreated fish, except in response to treatment with 250 μM Nefiracetam (Fig. S4). We tested whether a 1-h incubation would be sufficient to observe any effect on turning angles and found no statistically significant differences in the turning angles between treated and untreated animals (Fig. S5). We also tested five doses of (R)-Baclofen, 75 μM, 125 μM, 250 μM, 500 μM and 1 mM. Turning angles of wild-type animals did present a statistical significant difference at 75 μM, 250 μM and 500 μM, and turning angles of treated *myo7aa^−/−^* mutants animals were statistically significant compared with those of untreated animals at all of the doses (Fig. S6). We tested 1-h incubation of 125 μM (R)-Baclofen and identified this shorter incubation to be sufficient in decreasing the turning angles in the *myo7aa^−/−^* mutants (Fig. S7). We did not observe a statistical significance between untreated wild-type animals and those treated with 125 μM (R)-Baclofen after the 1-h incubation (Fig. S7).

Last, we assessed whether there would be a change in swim bladder inflation because the swim bladder is deflated in the *myo7aa*^−/−^ mutants. We did not observe any changes in swim bladder inflation when *myo7aa^−/−^* mutants or wild-type animals were incubated with 250 μM Nefiracetam or 125 μM (R)-Baclofen (Fig. S8).

### L-type voltage-gated calcium channel agonists increase acoustic startle response in *myo7aa*^−/−^ mutants

The *myo7aa^−/−^* mutant has little to no acoustic startle response to sounds between 200 Hz and 600 Hz (Fig. 7E). We hypothesized that we can reconstitute a functional response to sound by increasing the sensitivity of the calcium channel through treatment with L-type voltage-gated calcium channel agonist, thus augmenting the downstream signal in *myo7aa^−/−^* mutant hair cells. Wild-type fish treated with 5 μM (±)-Bay K 8644 had decreased responses at all frequencies, except 200 Hz (Table S5). Incubation with 125 μM (R)-Baclofen also decreased acoustic startle responses at all frequencies in wild-type fish (Fig. 7D; Table S5). However, wild-type fish treated with 250 μM Nefiracetam showed increased acoustic startle responses at all frequencies (Fig. 7D; Table S5). Both 5 μM (±)-Bay K 8644 and 250 μM Nefiracetam increased the acoustic startle response in the *myo7aa^−/−^* mutant fish. (±)-Bay K 8644 had a more robust effect and increased responses at all frequencies. However, we did not see any improvement of the acoustic startle response *myo7aa^−/−^* mutants upon incubation with 125 μM (R)-Baclofen (Fig. 7E; Table S5).

## DISCUSSION

In this study, we tested whether L-type voltage-gated calcium channel agonists can restore the abnormal synaptic morphological and behavioral phenotypes observed in the *myo7aa^−/−^* mutant to more closely resemble those of wild-type fish. The rationale for this hypothesis stems from understanding that the *myo7aa^−/−^* mutant cannot correctly gate the MET channel to reach the membrane potential required for depolarization. As a result, the L-type voltage-gated calcium channel does not open and synaptic transmission does not reach the level needed to detect meaningful sound. We analyzed whether the functional response to sound can be reconstituted by increasing the sensitivity of Ca_v_1.3 through treatment with L-type voltage-gated calcium channel agonists, thus, augmenting any residual signal in *myo7aa^−/−^* mutant hair cells. The agonists used were (±)-Bay K 8644, Nefiracetam and (R)-Baclofen. We identified the following: (1) comparable ribbon density area in *myo7aa^−/−^* mutants was observed by TEM; however, these ribbon densities harbor significantly fewer tethered vesicles. (2) (R)-Baclofen increased ribbon area and number of tethered vesicles. (3) As determined by immunohistochemistry, *myo7aa^−/−^* mutants have significantly fewer ribbons – with fewer Ctbp2 puncta in total; moreover, compared with those of wild-type fish, their distribution of Ctbp2 puncta was different. (4) All three compounds changed the distribution of Ctbp2 puncta to resemble that in wild-type fish. (5) The *myo7aa^−/−^* mutants have significantly fewer postsynaptic densities, although incubation with (±)-Bay K 8644 increased their number. (6) Turning angles of *myo7aa^−/−^* mutants were larger compared with those of wild-type fish. (7) All three L-type voltage-gated calcium channel agonists improved swimming behavior by decreasing the turning angles. (8) Treatment with (±)-Bay K 8644 and Nefiracetam improved the acoustic startle response in *myo7aa^−/−^* mutants. These results demonstrate that defects in the mechanotransduction apparatus affect the synaptic machinery but do not obliterate the ability of the cell to engage in synaptic activity. Further, we have shown that inner ear hair cells of the *myo7aa^−/−^* mutant are responsive to pharmacological rescue that translates into improved acoustic startle and changes in synaptic machinery.

### Synaptic morphologic differences in the *myo7aa*^−/−^ mutant can be modified by L-type voltage-gated calcium channel agonists

Ribbon synapse structures are found in hair cells and photoreceptor cells of vertebrate animals. They are specialized presynaptic synapses containing an electron-dense structure that tethers synaptic vesicles near voltage-gated calcium channels at the active zone of the hair cell. The main protein component of a ribbon synapse is Ribeye (Ctbp2), surrounded by a monolayer of tethered glutamatergic synaptic vesicles (Schmitz et al., 2000). It has previously been reported that hair cells of zebrafish larvae typically contain 3-5 ribbons (Nicolson, 2015; Obholzer et al., 2008). By using TEM, we observed that synaptic ribbons comprising an intact ultrastructure are localized correctly in the *myo7aa^−/−^* mutants, indicating that the machinery to generate and localize the ribbons is functional. It has also been reported that hair cell innervation is important for the correct localization of the ribbons at the plasma membrane, and for the regulation of ribbon size and number (Suli et al., 2016). Suli et al. also concluded that the physical presence of afferent fibers is enough to regulate ribbon formation. Therefore, our observation of the presence of ribbon structures at the synapse in *myo7aa^−/−^* mutants indicates that the hair cell is capable to stimulate afferent fibers and has the potential to respond to pharmacologic therapy.

We demonstrated here that (R)-Baclofen restores ribbon area and number of tethered vesicles in the *myo7aa^−/−^* mutant ribbon synapse. Incubation with (±)-Bay K 8644 and Nefiracetam did not influence ribbon area and number of tethered vesicles. Therefore, the mechanism by which (R)-Baclofen acts on the *myo7aa^−/−^* mutant ribbon synapses to increase ribbon area and number of tethered vesicles may not be directly and exclusively attributed to agonistic action of Ca_v_1.3. Nonetheless, our study is the first to describe the phenotype of ribbon synapses in the *myo7aa^−/−^* mutants and demonstrate that these ribbon synapses are altered in response to incubation with L-type voltage-gated calcium channel agonists to more closely resemble those of wild-type fish. However, ribbon synapses in wild-type fish remained unchanged upon incubation with any of the three agonists.

Ribeye is a splice isoform of Ctbp2 and is the central component of the ribbon synapse (Sheets et al. 2011; Wan et al. 2005). In zebrafish, Ribeye is essential to cluster L-type voltage-gated calcium channels to the active zone of the hair cell and these channels are found to regulate ribbon size and number (Sheets et al., 2011, 2012). This was also described in mouse cochlear hair cells (Frank et al., 2010). We explored the distribution of Ctbp2 puncta in *myo7aa^−/−^* mutants and discovered significantly fewer ribbon-containing cells – defined as those containing at least one Ctbp2 punctum – fewer Ctbp2 puncta in total and a different distribution of Ctbp2 puncta compared to those in wild type. We identified that most wild-type hair cells contain three Ctbp2 puncta and that most *myo7aa^−/−^* mutant hair cells have two Ctbp2 puncta. Our data showed that incubation with L-type voltage-gated calcium channel agonists shifts the distribution of Ctbp2 puncta and restores their distribution to resemble that in wild-type fish. This is an important finding, because it indicates that the key players involved in the regulation of Ctbp2 puncta are responding to pharmacological treatment.

It is well established that pre-synaptic cell activity affects postsynaptic cell activity and, conversely, postsynaptic cell activity can affect pre-synaptic cell activity (Luscher et al., 2000; Matus, 1999; Muller, 1997). Therefore, we use the postsynaptic cell marker MAGUK to assess the postsynaptic density. MAGUKs are members of a kinase superfamily, which target and anchor glutamate receptors to the synaptic terminal (Oliva et al., 2012). We identified that *myo7aa^−/−^* mutants have significantly fewer postsynaptic densities and fewer total MAGUK puncta per neuromast. Upon incubation with (±)-Bay K 8644 the number of postsynaptic densities and the total MAGUK puncta per neuromast increased in the *myo7aa^−/−^* mutants. We also quantified the number of cells with one, two, three, four, five and six+ MAGUK puncta, and found that there was no statistical difference between the distribution of MAGUK puncta in wild-type fish and *myo7aa^−/−^* mutants. We did not observe any effect in *myo7aa^−/−^* mutant neuromasts on the distribution of MAGUK puncta upon incubation with any of the compounds.

### Behavioral differences in the *myo7aa^−/−^* mutant can be mitigated by L-type voltage-gated calcium channel agonists

We utilized a quantitative behavior assessment that tracks zebrafish swimming in response to a static stimulus. We quantified the turning angle (absolute smooth orientation), which is the sum of all turning angles a fish swam in one swimming episode as a function of time. We identified that the global change in body orientation of *myo7aa^−/−^* mutants was larger than wild-type animals. We showed improved swimming behavior in the *myo7aa^−/−^* mutants in response to all three L-type voltage-gated calcium channel agonists. The most robust response was after treatment with (±)-Bay K 8644, which resulted in turning angles indistinguishable from those of wild type. Swimming trajectories of individual fish from each group also demonstrated less circular and looping swimming. We tested all three drugs in wild-type animals and observed no global change in body orientation at these specific doses, except for (±)-Bay K 8644. One possible explanation is that the Nefiracetam and (R)-Baclofen doses used were selected on the basis of dose-response studies (Figs S4 and S6) that had observed changes in turning angles of wild-type larvae to be dependent on the dose tested. We did not conduct a dose-response study for (±)-Bay K 8644.

The acoustic startle response to pure-tones can be elicited, starting at 5 dpf (Bhandiwad et al., 2013; Eaton and Didomenico, 1986; Zeddies and Fay, 2005). Saccular hair cells respond to relatively high frequencies (200-1200 Hz) and fibers that innervate the saccule synapse on Mauthner cells (Bang et al., 2002; Fay, 1995; Lin and Faber, 1988; Zottoli, 1977). The lateral line responds to much lower frequencies (50-100 Hz) (Coombs, 1994; Coombs and Janssen, 1990). We administered frequencies between 200 and 600 Hz to 5 dpf larval fish and recorded the absence or presence of an acoustic startle response. By using this assay, we identified that untreated wild-type fish exhibit the most robust responses at 200 Hz, which aligns with previous work by Bhandiwad et al. reporting the most sensitive response from 90-310 Hz (Bhandiwad et al., 2013). Incubation with (±)-Bay K 8644 and (R)-Baclofen decreased the mean response rate in the wild-type fish, but incubation with Nefiracetam improved the mean response rate.

Nicolson et al. reported that only 36% of hair bundles are splayed in the *myo7aa^−/−^* mutant; therefore, well over 50% of the stereocilia bundles are still intact (Nicolson et al., 1998). The authors also reported that the resting potential of hair cells remains within the range obtained for those of wild-type fish and that the stereocilia defects in the *myo7aa^−/−^* mutant are not due to an absence of driving force to transduce current. Last, they observed a small startle reflex calcium signal in the *myo7aa*^−/−^ mutant (Nicolson et al., 1998). All of this pivotal work indicates that the *myo7aa^−/−^* mutant is not starting at a zero response and, given that a majority of the hair bundles are still intact, the hair cell could potentially signal to the afferent nerve in response to sound. Our data showed that incubation with (±)-Bay K 8644 and Nefiracetam improved the acoustic startle response in the *myo7aa^−/−^* mutants. This is an important finding because it is the first report of a pharmacotherapy modulating acoustic startle in a zebrafish model of human Usher syndrome type 1. We concluded that restoration of the synaptic machinery by using L-type voltage-gated calcium channel agonists allowed the hair bundle as a whole to detect sound, as observed in our hearing assessment. Although our hearing assessment displayed a modest increase in acoustic startle responses, it is important to note that the startle response only tests the grossest aspects of hearing and, therefore, may present a high rate of Type II errors (i.e. false negatives), meaning that the auditory stimulus is detected but is too weak to elicit a startle response (Bhandiwad et al., 2013).

### Different effects of (±)-Bay K 8644, Nefiracetam and (R)-Baclofen on wild-type and *myo7aa*^−/−^ mutant hair cells

All three compounds utilized in this study target Ca_v_1.3; however, this is not the only mechanism of action for Nefiracetam and (R)-Baclofen. To understand the other possible targets of these compounds will elucidate the effects they have on wild-type and *myo7aa^−/−^* mutant hair cells. (±)-Bay K 8644 is a potent L-type calcium channel activator (Nowycky et al., 1985; Schramm et al., 1983). It was used extensively to study regulation of calcium influx and the role of L-type calcium channels in synaptic transmission in neuronal cells (Bonci et al., 1998; Monjaraz et al., 2000; Watanabe et al., 1998). Nefiracetam is a nootropic or cognitive enhancer that activates both L and N-type calcium channels (Yoshii and Watabe, 1994; Yoshii et al., 2000), and may modulate the GABA receptor currents by interacting with a PKA pathway (Huang et al., 1996) and PKC pathway (Nishizaki et al., 1998). Nootropic agents can influence various neurotransmissions, including dopaminergic, cholinergic, glutamatergic and GABAergic pathways (Funk and Schmidt, 1984; Giovannini et al., 1993; Huang et al., 1996; Marchi et al., 1990; Nabeshima et al., 1990; Nishizaki et al., 1998; Oyaizu and Narahashi, 1999; Watabe et al., 1993). Additionally, Nefiracetam affects acetylcholine-induced currents and NMDA receptors (Moriguchi et al., 2003; Narahashi et al., 2003; Zhao et al., 2001). NMDA and AMPA receptors are ionotropic glutamate receptors that are nonselective cation channels (K^+^ and Na^+^). Activation of these channels produces excitatory postsynaptic responses. NMDA receptors allow entry of potassium, sodium and calcium ions; this increase in intracellular calcium acts as a secondary messenger to augment intracellular signaling cascades. Last, (R)-Baclofen acts as a GABA_B_ agonist (Bowery et al., 1983). Recently it has been reported that GABA_B_ receptors modulate several voltage-gated calcium channels (Carter and Sabatini, 2004; Shen and Slaughter, 1999). Park et al. has identified a direct interaction between the L-type voltage-gated calcium channel (Ca_v_1.3) and the GABA_B_ receptor subunit 2, and that activation of the GABA_B_ receptor increases L-type calcium currents (Park et al., 2010).

We identified that (±)-Bay K 8644 significantly increased the total numbers of ribbon-containing cells, Ctbp2 puncta, postsynaptic densities and MAGUK puncta per neuromast in wild-type animals. We also observed that, upon incubation with 5 μM (±)-Bay K 8644, the distribution of Ctbp2 puncta shifted in wild-type larvae such that most hair cells showed two Ctbp2 puncta. Additionally, 5 µM (±)-Bay K 8644 decreased the turning angles and acoustic startle response in wild-type animals. Only (±)-Bay K 8644 is likely to have all these effects on wild-type animals because it is the most potent of the three compounds and its only known mechanism of action is on the Ca_v_1.3 channel. Another possible explanation is that we are observing a bell-shaped dose response curve, in which drug efficacy increases with drug concentration to a maximum level, above which the effect is reduced. This effect is reported in over 1000 citations to molecules in the literature (Owen et al., 2014). This can be tested by re-examining the effect of (±)-Bay K 8644 on all assessments with higher and lower doses of the drug. Another possible explanation is that the changes in calcium concentration induced by (±)-Bay K 8644 in a normal hair cell – away from homeostasis – can have consequential effects. However, in the hair cell of a *myo7aa^−/−^* mutant that already has a low initial calcium concentration (below the level of homeostasis) pharmacologic strategies to increase intracellular calcium may bring the calcium concentration to a level closer to normal and provide a therapeutic effect.

Nefiracetam and (R)-Baclofen have other mechanisms of action besides targeting Ca_v_1.3. Therefore, to undoubtedly attribute the effects of these compounds to agonistic activity on Ca_v_1.3 would require additional investigation. Furthermore, possible unexpected effects on wild-type animals may be attributed to other mechanisms of action. We observed that (R)-Baclofen increased the number of postsynaptic densities and total number of MAGUK puncta in wild-type larvae. This could be due to the GABA_B_ agonistic activity of (R)-Baclofen (Bowery et al., 1983). It has been reported that incubation with (R)-Baclofen can have a sedative effect in zebrafish, which decreases locomotor activity and is dose dependent (Renier et al., 2007). Renier et al. have reported that doses as low as 10^−4^ M decreased the locomotor activity to ∼25-30% of that observed in controls. In our study, we observed that wild-type fish treated with 125 µM (R)-Baclofen exhibits ∼20% of control fish mean response rate at each individual frequency. We did not observe this sedative effect in the swimming behavior assessment, for which the stimulus was a static shock. Tabor et al. have reported that electric field pulses do not require synaptic transmission to stimulate the Mauthner cells (Tabor et al., 2014). Therefore, any sedative effect on the fish due to incubation with 125 µM (R)-Baclofen would be overridden by the ultrarapid response to an electric stimulus.

### Conclusion

Here, we report for the first time the use of L-type voltage-gated calcium channel agonists to treat the zebrafish model of USH1, the *myo7aa^−/−^* mutant. We have shown through TEM and immunohistochemistry that the synaptic machinery of the *myo7aa^−/−^* mutant differs to that of wild-type in that there are fewer glutamatergic vesicles tethered to the ribbon density, fewer total ribbon-containing cells and postsynaptic densities, fewer total Ctbp2 and MAGUK puncta, and a different distribution of Ctbp2 puncta. We quantified the abnormal swimming behavior and identified that *myo7aa^−/−^* fish show larger turning angles. Upon treatment with L-type voltage-gated calcium channel agonists, we discovered changes in hair cell ultrastructure, Ctbp2 protein distribution as well as in behavior of fish through swimming and hearing assessments. Our data show that treatment with these compounds restores the abnormalities in *myo7aa^−/−^* mutants, so they more closely resemble wild-type fish. We found that treatment (1) increased the number of tethered vesicles to the ribbon density; (2) shifted the distribution of Ctbp2 puncta; (3) decreased turning angles, thus, improving swimming behavior and; (4) improved the acoustic startle response. However, there are still unanswered questions regarding the exact mechanisms of action for each agonist and which of these pathways provide a therapeutic effect. We are also interested in exploring any mitochondrial contribution to both the phenotype observed in the mutant, and to the changes elicited in the synaptic components and behavior upon incubation with these compounds. Moreover, direct quantification of calcium concentrations with and without treatment is necessary to firmly conclude whether calcium is driving the changes at the synapse. We are confident that our results here represent a significant step towards the understanding of the mechanism of the disease and the discovering of compounds to effectively treat hearing loss caused by pathogenic variants in *MYO7A*.

## MATERIALS AND METHODS

### Zebrafish maintenance and husbandry

All animals used in this study were zebrafish (*Danio rerio*). The zebrafish strains used for all experiments were the *mariner* tc320b allele (c.2699T>A; p.Tyr846Stop) in exon 21 of *myo7aa* (Ernest et al., 2000), wild-type Segrest strains were obtained from the Mayo Clinic laboratory stock (Leveque et al., 2016). Adult fish heterozygous for the *myo7aa* mutation were used to generate homozygous larval fish (*myo7aa^−/−^*). Animals were raised in a 10-h dark and 14-h light cycle, with embryos and adults kept at 28.5°C (Westerfield, 1995). Embryos were kept in Petri dishes and embryo water was replaced daily. Larvae were kept at room temperature for all experiments and maintained in the incubator all other times. Wild-type Segrest strains and heterozygous adult *myo7aa* fish were also maintained at the University of Minnesota, Twin Cities Zebrafish Core Facility. The Institutional Animal Care and Use Committee approved all experimental procedures both at Mayo Clinic and the University of Minnesota.

### Phenotyping and genotyping

The *myo7aa^−/−^* tc320b allele results from a single A-T nucleotide change, yielding an early stop codon p.Tyr846Stop (Ernest et al., 2000). Homozygous recessive mutant larval fish were identified at 4 days post fertilization (dpf) based on various phenotypic traits, including defective balance, absent startle to sound and circular or looping swimming patterns. DNA extracted from whole embryos or fin biopsies was used for allele-specific quantitative PCR and genotyping as previously described (Lee et al., 2016).

### Hair cell staining

To stain the stereocilia of the saccule hair cells, embryos were initially fixed overnight in freshly thawed 4% paraformaldehyde at 4°C. Two washes for 5 min each in 0.2% PBSTx (1× PBS with 0.2% Triton X-100) were conducted at room temperature. Embryos were then incubated for 4 days in 2% PBSTx at 4°C to completely dissolve the otoliths. F-actin staining occurred for up to 2 h at room temperature when using Alexa-Fluor-488 tagged to phalloidin (Thermo Fisher Scientific, Eugene, OR, USA), which was diluted 1:20 in 1×PBS with Tween20 (PBSTw). Embryos were then washed six times for 10 min at room temperature in 0.2% PBSTx. Hair cell stereocilia were observed and imaged by using an LSM 780 inverted confocal microscope run and Zen software package (Zeiss).

### MET channel assessment

Mechanoelectrical transduction (MET) channel activity was assessed through uptake of FM 1-43 dye (Thermo Fisher Scientific). A working stock of 3 µM FM 1-43 was used for a 30 s incubation and rinsed three times immediately with embryo water. Lateral line neuromasts were imaged for FM 1-43 uptake using a Lightsheet Z.1 microscope (Zeiss). Lateral-orientated *z*-stacks at 5×/1.0 NA water-dipping objective (Zeiss) and an RFP (emission filter BP 505-545 LP 585) optical filter (Zeiss). Each corresponding dorsal image is a maximal image projection generated from *z*-dimension stacks.

### Drug incubation

The maximum tolerated dose was determined for all drugs used in this study. This was done by individually incubating 4 dpf wild-type embryos within 24-well plates in 1 ml total volume of embryo water, including various doses of dissolved drug. Control fish were incubated in the same concentration of embryo water and drug vehicle, e.g. DMSO, H_2_O only. The 24-well plates were placed in a 29°C embryo incubator for the duration of drug treatment. Heart rates, startle responses, swimming behavior and overall health were observed after incubation for 1 h, 4 h and overnight, to determine any adverse effects or possible toxicity. Drug doses started at 0.5 μM and increased, depending on overall health and survival of the embryos. Drugs used in this study were as follows: (±)-Bay K 8644 (catalog #1544, Batch No. 4; Tocris), Nefiracetam (catalog #2851, Batch No. 1; Tocris) and (R)-Baclofen (catalog #0796, Batch No. 3; Tocris). (±)-Bay K 8644 and Nefiracetam were dissolved in DMSO to a 100 mM stock solution, (R)-Baclofen was dissolved in water (with gentle warming) to a 20 mM stock solution. The final doses used for drug treatments were determined by identifying the highest tolerated dose and using the second-highest dose as the final dose. Control experiments were conducted to determine effects of DMSO on behavior and the concentrations used for dissolving drugs. No adverse effects were identified. Animals were randomly assigned to either control or drug-treated groups within each genotype.

### Video acquisition of swimming behavior

Videos were made according to the video acquisition protocol previously described (Lambert et al., 2012). High-speed videos of 5 dpf larvae were acquired at either 120 or 180 frames per second (fps) using FlyCap software and the Point Grey Firefly. A LED light stage was used underneath a 20 mm area to increase illumination and improve video quality. A 50 V electric shock (5 ms duration) was used to startle the animal every 20 s. An LED light was used to confirm administration of the electric shock.

### Video tracking and analysis

Videos acquired by using the FlyCap software were processed as fmf files through Ctrax: The Caltech Multiple Fly Tracker, free source software (Branson et al., 2009). The tracking settings for motion, observation and hindsight are listed in Table S1. Videos were exported as mat files, fixed for errors by applying the Fix Errors MATLAB Toolbox (FEMT), provided by Branson et al. (2009), and analyzed in MATLAB version 2012b by using in-house scripts from the Masino Laboratory (available upon request). Swimming trajectories were generated by using the Ctrax Behavioral Microarray MATLAB toolbox. A circle indicating the perimeter of the testing well was added to each image as a reference point. Each electric shock was confirmed by determining the frame in which the LED light appears when using ImageJ. The frame range corresponding to that particular swimming episode was found in the mat files. The values for the absolute smooth orientation of the fish during one swimming episode were totaled and then divided by the episode duration. This was performed for all swimming episodes. Outlier analysis was conducted on each individual experiment and outliers were excluded from the final data set.

### Hearing assessment

Up to ten 4 dpf embryos were placed in 60×15 mm Petri dishes and placed in a 29°C embryo incubator in an isolated behavior room for overnight incubation. At 5 dpf, embryos were placed on top of a Bose Soundlink Color Speaker and given ≤5 min to acclimate. The speaker was placed on top of an illuminator for a better quality video. A white piece of paper was placed beneath the Petri dish to lighten the background in order to create contrast between the larval fish and the speaker. A script written in MATLAB (available upon request) was used to generate three 1-s stimuli every 20 s for frequencies of 200, 300, 400, 500 and 600 Hz, and administered via Bluetooth connection between laptop and speaker. The intensity of the sounds administered was determined using an Extech sound level meter, at ∼77 dB. Videos were taken with a Sony Handycam HDR cx560, and de-identified and blindly scored by a trained laboratory member for startle responses. Responses were scored as either 0 (no response) or 1 (response) for each fish at each stimulus. Mean values of responses and no responses were calculated, and mean variances determined.

### Processing and imaging of zebrafish for TEM

5 dpf embryos were fixed in Trump's fixative (4% paraformaldehyde with 1% glutaraldehyde) for a minimum of 24 h. Following fixation, embryos were fixed again with 1% osmium tetroxide and 2% uranyl acetate, dehydrated through an ethanol series and embedded into Embed 812/Araldite resin. Processing was facilitated by using a BioWave laboratory microwave oven (Pelco Biowave 3450, Ted Pella, Inc., Redding, CA, USA). After 24 h polymerization at 60°C, ultrathin sections (0.1 mm) were stained with 2% uranyl acetate and lead citrate. Micrographs of synaptic ribbons from inner ear hair cells were acquired in the Mayo Clinic Microscopy and Cell Analysis Core using a JEOL1400 or JEOL1400Plus transmission electron microscope operating at 80 kV (Peabody, MA) equipped with a Gatan Orius camera (Gatan, Inc., Warrendale, PA, USA).

### Analysis of ribbon synapse TEM images

Transmission electron microscopy (TEM) images of ribbon synapse structures from the inner ear were de-identified and blindly analyzed for ribbon area and number of tethered vesicles. A region of interest (ROI) was drawn around the electron dense structure or the central component of the ribbon using ImageJ. Each ROI and the number of vesicles tethered to this central component were quantified. Images were re-identified after completion of the analysis.

### Immunohistochemistry

Preparation for and carrying out of immunohistochemistry (IHC) experiments were conducted over 5 days by using a nutator unless otherwise specified. The first day included fixation 5 dpf embryos by overnight incubation in 4% paraformaldehyde at 4°C. Samples were then rinsed twice and washed three times for 10 min each with PBTr (0.02% Triton-X 100 in 1×PBS), washed for 5 min in a 1:1 mixture (PBTr and 100% MetOH) and then washed three times for 5 min in 100% MetOH. Samples were used immediately or stored at −20°C. The third day included rehydrating the embryos through a 5-min wash of the 1:1 mixture followed by three 5-min washes with PBTr. Samples were permeabilized with proteinase K (diluted 1:2000 in PBTr) for 50 min and then washed five times for 5 min with PBTr. Block buffer included 10% normal goat serum, 0.2% Triton-X 100, 2% DMSO in 1×PBS. Samples were incubated in block buffer for ≥3 h. The primary antibodies used in this study were mouse monoclonal anti-pan MAGUK; 1:250 NeuroMab Clone K28/86, Catalog #75-029, Lot #449-5AK-12b) (Anderson, 1996; Lv et al., 2016; Sebe et al., 2017; Sheets, 2017; Sheets et al., 2011, 2017) and anti-Ctbp2 (1:500 Santa Cruz Catalog #Sc-55502, Lot #C2816) (Kindt and Sheets, 2018) diluted in prepared block buffer. Samples were incubated in diluted primary antibody at 4°C overnight. The fourth day included washing samples six times for 30 min with PBTr. Secondary antibodies used were Alexa-Fluor-647 goat anti-mouse (1:250 Thermo-Fisher/Invitrogen Catalog #A21235, Lot #890864) and Alexa-Fluor-488 goat anti-mouse (1:500 Thermo-Fisher/Invitrogen Catalog #A21121, Lot #1964382), both diluted in PBTr. Samples were incubated in diluted secondary antibody at 4°C overnight. Last, samples were washed again six times for 30 min with PBTr and washed three times for 5 min with 1×PBS. Samples were stored in 1×PBS at 4°C. Images were acquired using the LSM 780 inverted confocal microscope run on Zen software package (Zeiss). Samples were mounted in Vectashield Hardset DAPI Mounting Medium and covered with a cover slip for ∼10 min before imaging. We confidently report reproducibility of experiments, specificity – determined by lack of fluorescence in cells that should not contain the antigen of interest – as well as running appropriate controls (primary only and secondary only) for validation (Bordeaux et al., 2010). All images are of the MI1 neuromast and were captured with a 20× objective and a 5.0 magnitude zoom. All images were de-identified prior to analysis. Analysis was performed using ImageJ software. The proportion of ribbon-containing cells or postsynaptic densities with one, two, three, four, five or six+ puncta were blindly determined by identifying the number of cells with *n* puncta divided by the total number of ribbon ribbon-containing cells or postsynaptic densities in the MI1 neuromast. Images were re-identified after completion of the analysis.

### Statistical analysis

Statistical analyses were performed using JMP software (JMP^®^ 1989-2019). Each treatment group was individually compared to the corresponding control group through an unpaired two-tailed *t*-test. Sample size was calculated after initial control experiments, i.e. two independent samples, were conducted for a continuous outcome (*α*=0.05, *β*=0.2, *P*=0.8). Statistical analysis of the acoustic startle response was performed with GraphPad using two-tailed Fisher's exact test. All statistical analyses are presented with appropriate significant digits.

## Supplementary Material

Supplementary information
