# Supplementary material for: L-type voltage-gated calcium channel agonists mitigate hearing loss and modify ribbon synapse morphology in the zebrafish model of Usher syndrome type 1
Source: Dis Model Mech. 2020 Nov 27;13(11):dmm043885. doi: 10.1242/dmm.043885 (PMC7710014; doi:10.1242/dmm.043885)
Supplement: Supplementary information [file dmm-13-043885-s1.pdf]

**Setting**	**Value**
Angle Weight	100
Max Jump Distance	80
Max Jump D to Split	80
Min Jump Distance	10000
Center Dampening	0
Angle Dampening	0.5
Max Area Delete	200
Min Area Ignore	20000
Max Penalty Merge	5000
Lower Thresh	1
Max Clusters Per Blob	1
Max Blobs to Detect	2
Max Sequence Length	120 or 180 (frame rate)
Max Penalty Merge	5000
Max Pred Error Increase	5000

Table S1. Tracking settings for motion, observation, and hindsight in Ctrax: The Caltech Multiple Fly Tracker.

	**wildtype untreated**		**wildtype untreated**
CTBP2 Puncta	*myo7aa*$^{-/-}$ untreated	MAGUK Puncta	*myo7aa*$^{-/-}$ untreated
1	0.61	1	0.32
2	**0.05**	2	0.85
3	**0.02**	3	0.16
4	0.34	4	0.15
5	0.46	5	0.93
6+	0.07	6+	0.16

Table S2. *myo7aa*$^{-/-}$ have a statistically different distribution of CTBP2 puncta in the ribbon containing cells, but a similar distribution of MAGUK puncta in the post-synaptic densities. A t-test was used to determine statistical significance between wildtype and the *myo7aa*$^{-/-}$ mutant distribution of CTBP2 and MAGUK puncta. P-values are represented in each cell.

**A**	**wildtype untreated**		
CTBP2 Puncta	wildtype 5 µM (±)-Bay K 8644	wildtype 250 µM Nefiracetam	wildtype 125 µM (R)-Baclofen
1	**0.0003**	**0.64**	**0.61**
2	**<0.0001**	0.13	**0.01**
3	**0.04**	0.30	0.25
4	**0.03**	0.13	0.56
5	0.07	0.87	0.13
6+	0.18	0.38	0.04

**B**	***myo7aa***$^{-/-}$ **untreated**		
CTBP2 Puncta	*myo7aa*$^{-/-}$ 5 µM (±)-Bay K 8644	*myo7aa*$^{-/-}$ 250 µM Nefiracetam	*myo7aa*$^{-/-}$ 125 µM (R)-Baclofen
1	0.06	0.23	0.28
2	0.27	0.34	0.22
3	**0.036**	**0.048**	**0.025**
4	0.27	0.66	0.22
5	0.09	0.17	0.11
6+	0.63	0.57	0.74

Table S3. L-type voltage-gated calcium channel agonists change the distribution of CTBP2 puncta in *myo7aa*$^{-/-}$ mutant ribbon containing cells. Table A represents the statistical analysis for all wildtype treated animals compared to untreated wildtype animals for each number of puncta. (±)-Bay K 8644 had the largest effect on wildtype ribbon containing cells. Table B represents statistical analysis for all *myo7aa*$^{-/-}$ treated animals compared to untreated *myo7aa*$^{-/-}$ animals for each number of puncta. All three L-type voltage-gated calcium channel agonists had a statistical significance at 3 puncta. A t-test was used to determine statistical significance between treated and untreated groups. P-values are represented in each cell.

**A**	**wildtype untreated**		
MAGUK Puncta	wildtype 5 µM (±)-Bay K 8644	wildtype 250 µM Nefiracetam	wildtype 125 µM (R)-Baclofen
1	0.22	0.08	0.79
2	0.10	0.10	0.36
3	0.98	0.92	0.37
4	**0.03**	0.64	0.12
5	0.31	0.99	0.44
6+	0.67	0.60	0.08

**B**	***myo7aa***$^{-/-}$ **untreated**		
MAGUK Puncta	*myo7aa*$^{-/-}$ 5 µM (±)-Bay K 8644	*myo7aa*$^{-/-}$ 250 µM Nefiracetam	*myo7aa*$^{-/-}$ 125 µM (R)-Baclofen
1	0.83	0.74	0.45
2	0.32	0.68	0.26
3	0.46	0.64	0.40
4	0.65	0.66	0.61
5	0.98	0.78	0.33
6+	0.61	0.79	0.29

Table S4. L-type voltage-gated calcium channel agonists do not change the distribution of MAGUK puncta in *myo7aa*$^{-/-}$ mutant post-synaptic densities. Table A represents statistical analysis for all wildtype treated animals compared to untreated wildtype animals for each number of puncta. There was no change in the distribution of MAGUK puncta, except at 4 puncta upon incubation with 5 µM (±)-Bay K 8644. Table B represents statistical analysis for all *myo7aa*$^{-/-}$ treated animals compared to untreated *myo7aa*$^{-/-}$ animals for each number of puncta. All three L-type voltage-gated calcium channel agonists had no effect on the number of MAGUK puncta in the post-synaptic densities. A t-test was used to determine statistical significance between treated and untreated groups. P-values are represented in each cell.

**A**	**wildtype untreated**		
Frequency (Hz)	wildtype<br>5 µM (±)-Bay K 8644	wildtype<br>250 µM Nefiracetam	wildtype<br>125 µM (R)-Baclofen
200	0.34	**0.0001**	**<0.0001**
300	**0.0009**	**0.003**	**<0.0001**
400	**0.04**	**0.01**	**<0.0001**
500	**<0.0001**	**0.03**	**<0.0001**
600	**0.002**	**0.006**	**0.0004**

**B**	***myo7aa$^{-/-}$* untreated**		
Frequency (Hz)	*myo7aa$^{-/-}$*<br>5 µM (±)-Bay K 8644	*myo7aa$^{-/-}$*<br>250 µM Nefiracetam	*myo7aa$^{-/-}$*<br>125 µM (R)-Baclofen
200	**<0.0001**	**0.016**	1.0
300	**0.006**	1.0	1.0
400	**<0.0001**	**0.008**	1.0
500	**0.006**	0.08	1.0
600	**<0.0001**	0.14	0.51

Table S5. (±)-Bay K 8644 produces the most robust improvement in acoustic startle response in *myo7aa$^{-/-}$* mutants. Table A represents statistical analysis for all wildtype treated animals compared to untreated wildtype animals for each frequency. Table B represents statistical analysis for all *myo7aa$^{-/-}$* mutants treated compared to untreated *myo7aa$^{-/-}$* mutants for each frequency. A two-tailed Fisher's exact test was used to determine statistical significance between untreated and treated group. P-values are represented in each cell.

Disease Models & Mechanisms • Supplementary information

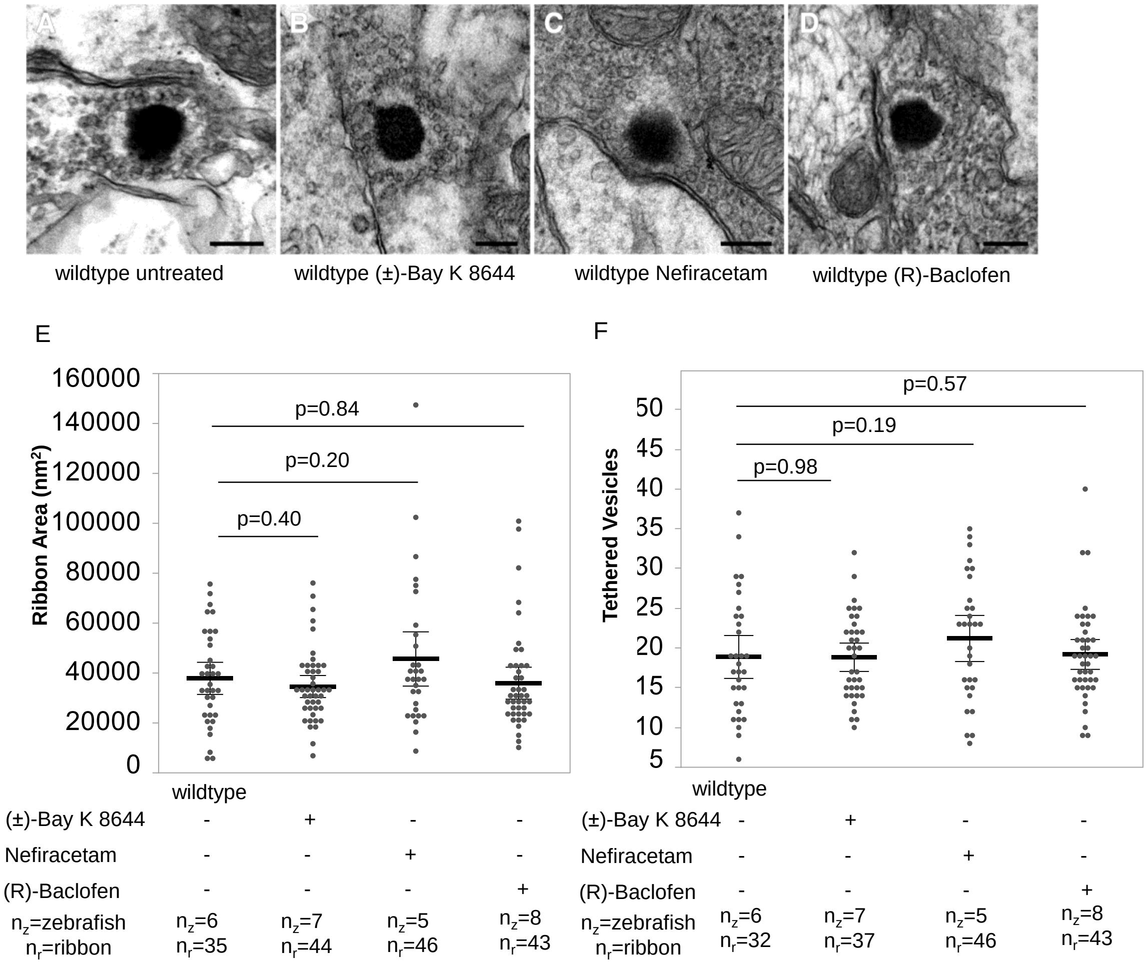

Fig. S1. Wildtype ribbon synapses unaffected with exposure to L-type voltage-gated calcium channel agonists. (A-D) Representative images of 5 dpf wildtype untreated and treated with 5 µM (±)-Bay K 8644 (B), 250 µM Nefiracetam (C) and 125 µM (R)-Baclofen (D) ribbon synapse structure obtained with transmission electron microscopy. Scale bars: 200 nm. (E) Wildtype treated and untreated ribbon synapse areas are comparable. (F) Additionally, untreated and treated wildtype ribbons have a comparable number of tethered vesicles. Black bold line represents the mean of the data set and error bars are 95% confidence intervals. Experiments were replicated two times for wildtype animals incubated in 5 µM (±)-Bay K 8644, four times for animals incubated in 250 µM Nefiracetam and three times for animals incubated in 125 µM (R)-Baclofen.

Disease Models & Mechanisms • Supplementary information

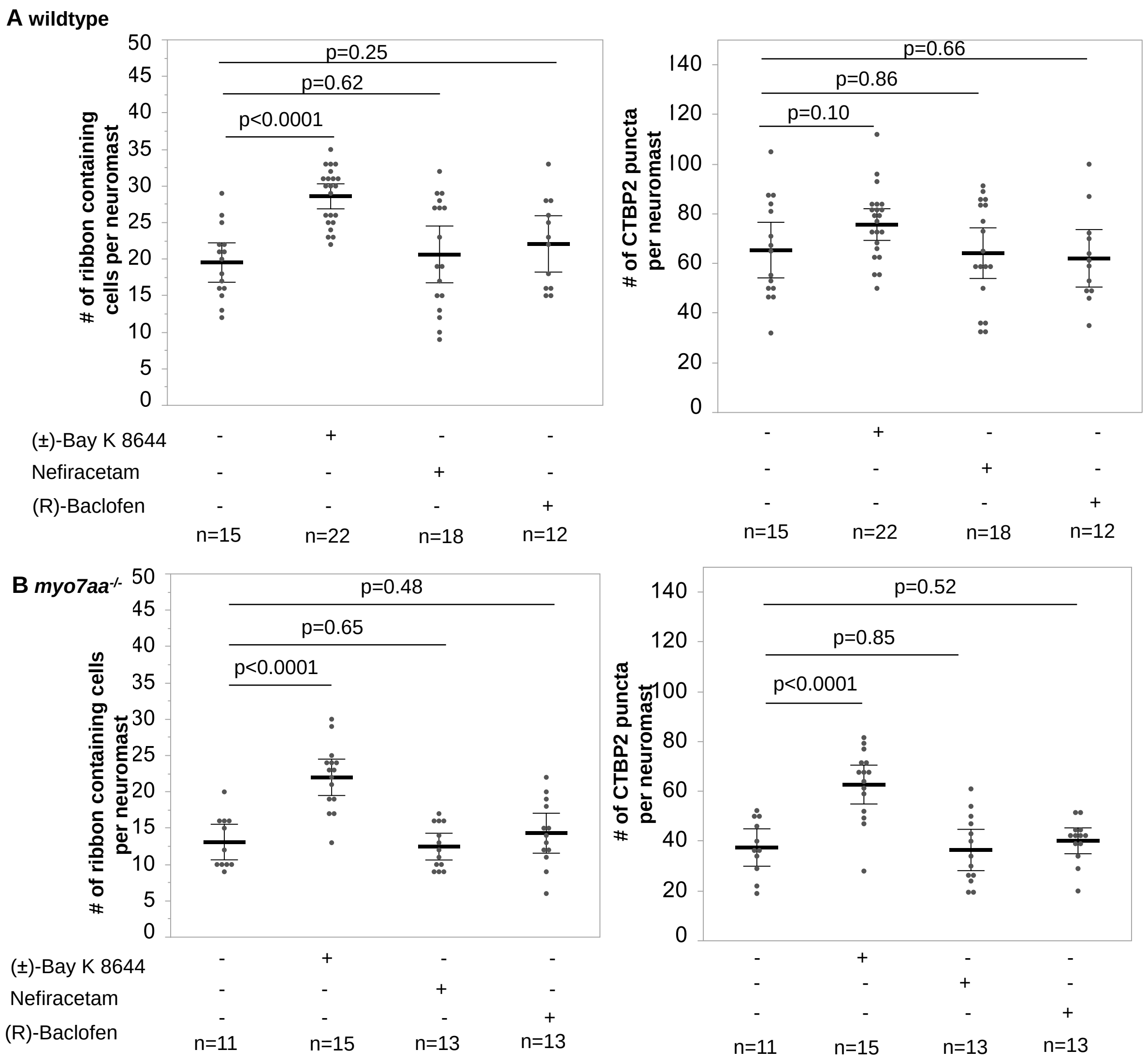

Fig. S2 (±)-Bay K 8644 increases total number of ribbon containing cells per neuromast and total number of CTBP2 puncta per neuromast in both wildtype and *myo7aa$^{-/-}$* mutants. (A) 5 dpf wildtype neuromasts treated with (±)-Bay K 8644 have a greater number of ribbon containing cells and total number of CTBP2 puncta compared to untreated wildtype neuromasts (t-test). (B) 5 dpf *myo7aa$^{-/-}$* neuromasts treated with (±)-Bay K 8644 have a greater number of ribbon containing cells per neuromast and total number of CTBP2 puncta compared to untreated *myo7aa$^{-/-}$* neuromasts (t-test). Black bold line represents the mean of the data set and error bars are 95% confidence intervals.

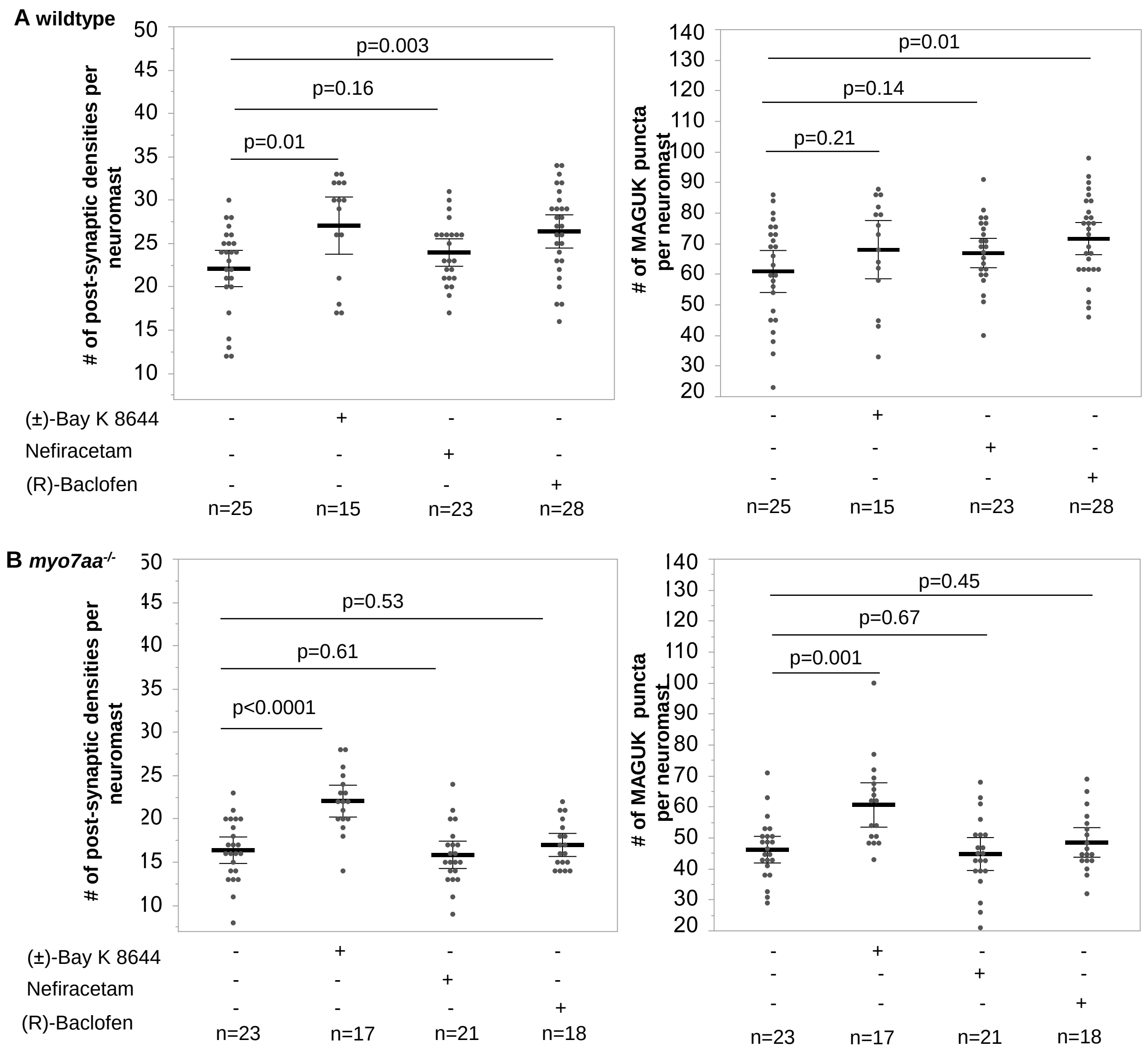

Fig. S3 (±)-Bay K 8644 increases total number of post-synaptic densities per neuromast and total number of MAGUK puncta per neuromast in the *myo7aa$^{-/-}$* mutants. (A) 5 dpf wildtype neuromasts treated with (±)-Bay K 8644 have a greater number of post-synaptic densities and those treated with (R)-Baclofen have an increase in both the number of post-synaptic densities and total number of MAGUK puncta compared to untreated wildtype neuromasts (t-test). (B) 5 dpf *myo7aa$^{-/-}$* neuromasts treated with (±)-Bay K 8644 have a greater number of post-synaptic densities and total number of MAGUK puncta compared to untreated *myo7aa$^{-/-}$* neuromasts (t-test). Black bold line represents the mean of the data set and error bars are 95% confidence intervals.

Disease Models & Mechanisms • Supplementary information

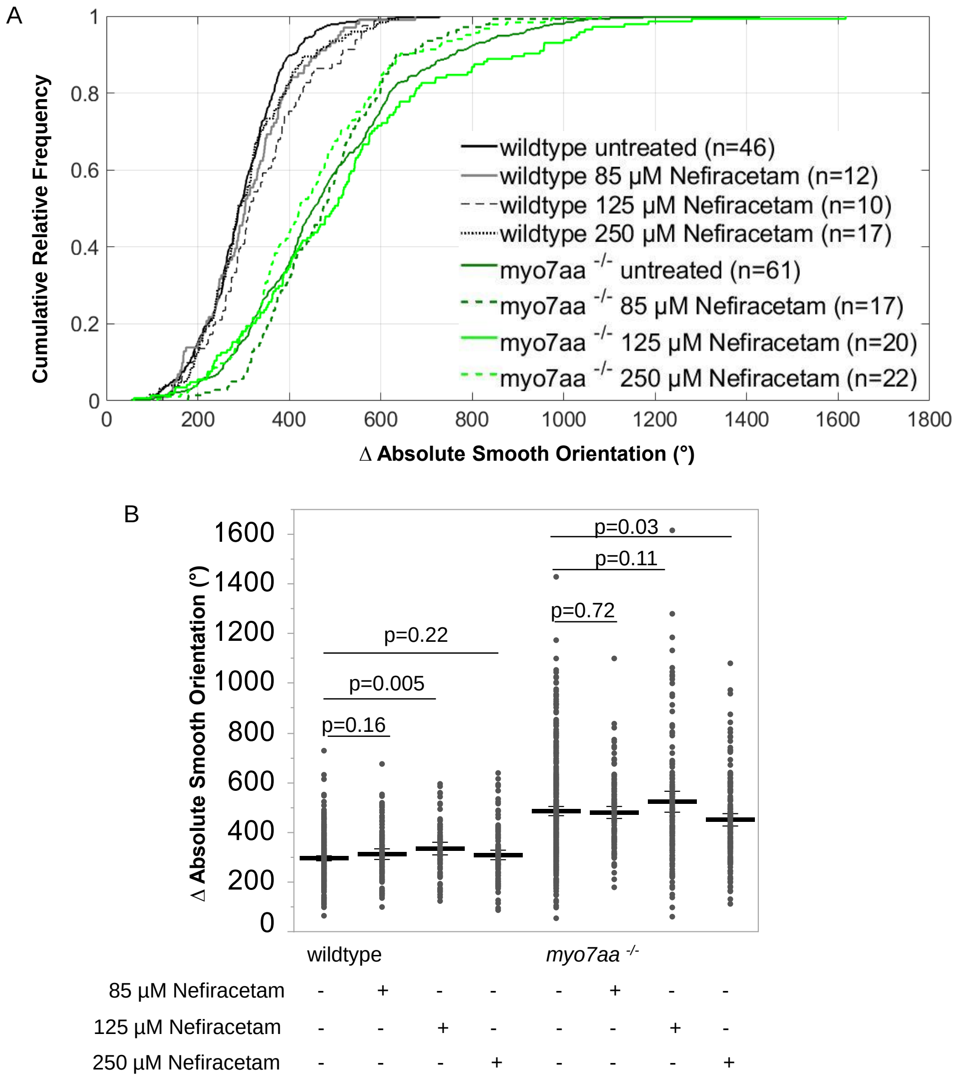

Fig. S4 Different doses of Nefiracetam have different effects on wildtype and *myo7aa$^{-/-}$* mutant swimming behavior. This behavior assessment tracks movement of 5 dpf wildtype and *myo7aa$^{-/-}$* larvae over a 2.5 minute interval with a 5 ms electric stimulus (50 mV) administered every 20 s. Ctrax software was used for video processing and Matlab 2012b for video analysis. (A, B) Incubation with 85 µM and 250 µM Nefiracetam had no adverse effects on wildtype swimming behavior; however incubation with 125 µM increased the absolute smooth orientation (turning angle as a function of time) of wildtype larvae. Incubation with 85 µM and 125 µM Nefiracetam had no effect on *myo7aa$^{-/-}$* larvae but incubation with 250 µM did decrease the absolute smooth orientations compared to those untreated. Individual turning angles from populations of wildtype and *myo7aa$^{-/-}$* larvae treated and untreated were used to construct the lines (t-test). The black bold line represents the mean of the data set and error bars are 95% confidence intervals. These experiments were replicated twice for each dose for each genotype.

Disease Models & Mechanisms • Supplementary information

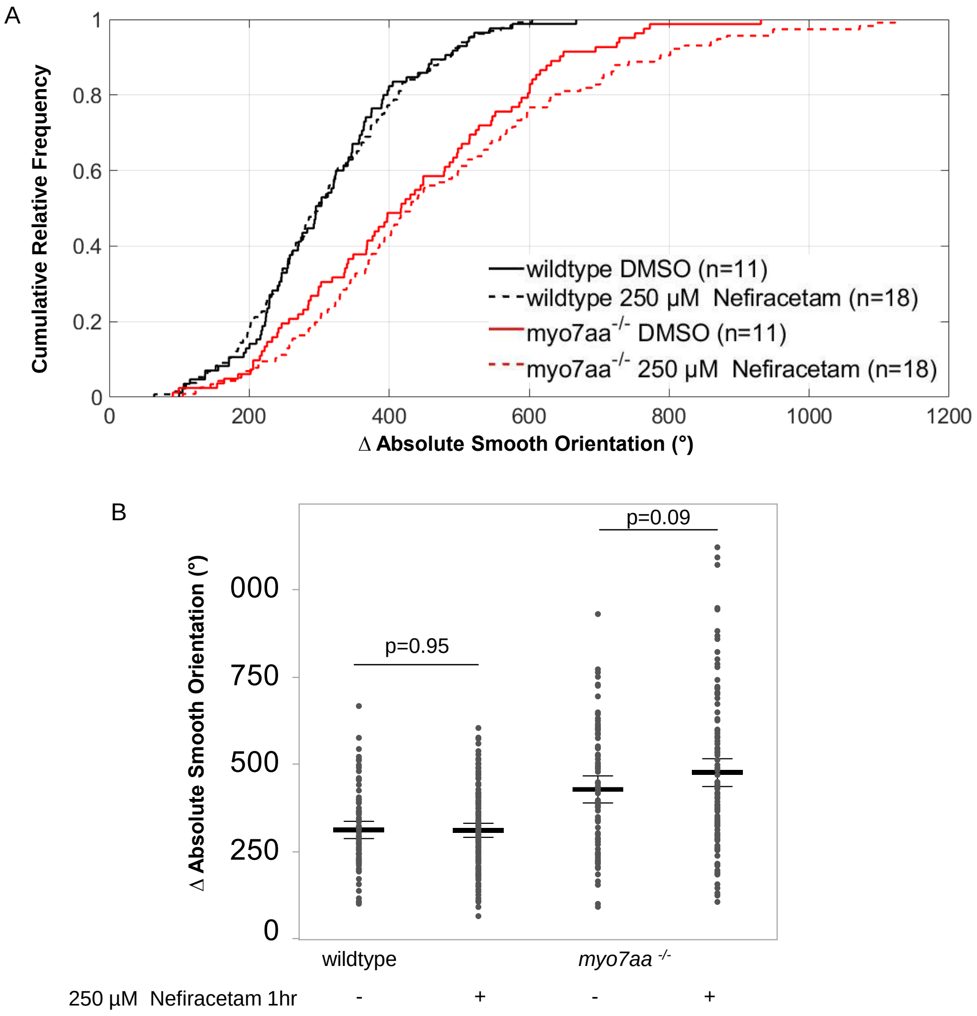

Fig. S5 1 hour incubation does not suffice for Nefiracetam to affect swimming behavior of wildtype and *myo7aa*$^{-/-}$ mutants. In order to determine if a shorter incubation period would provide similar responses to an overnight incubation the absolute smooth orientation was quantified. (A, B) Incubation with 250 µM Nefiracetam for 1 hour had no adverse effects on wildtype swimming behavior and did not affect the absolute smooth orientation of the *myo7aa*$^{-/-}$ mutants, indicating that an overnight incubation is required. Individual turning angles from populations of wildtype and *myo7aa*$^{-/-}$ larvae treated and untreated were used to construct the lines (t-test). The black bold line represents the mean of the data set and error bars are 95% confidence intervals. These experiments were replicated twice for each dose for each genotype.

Disease Models & Mechanisms • Supplementary information

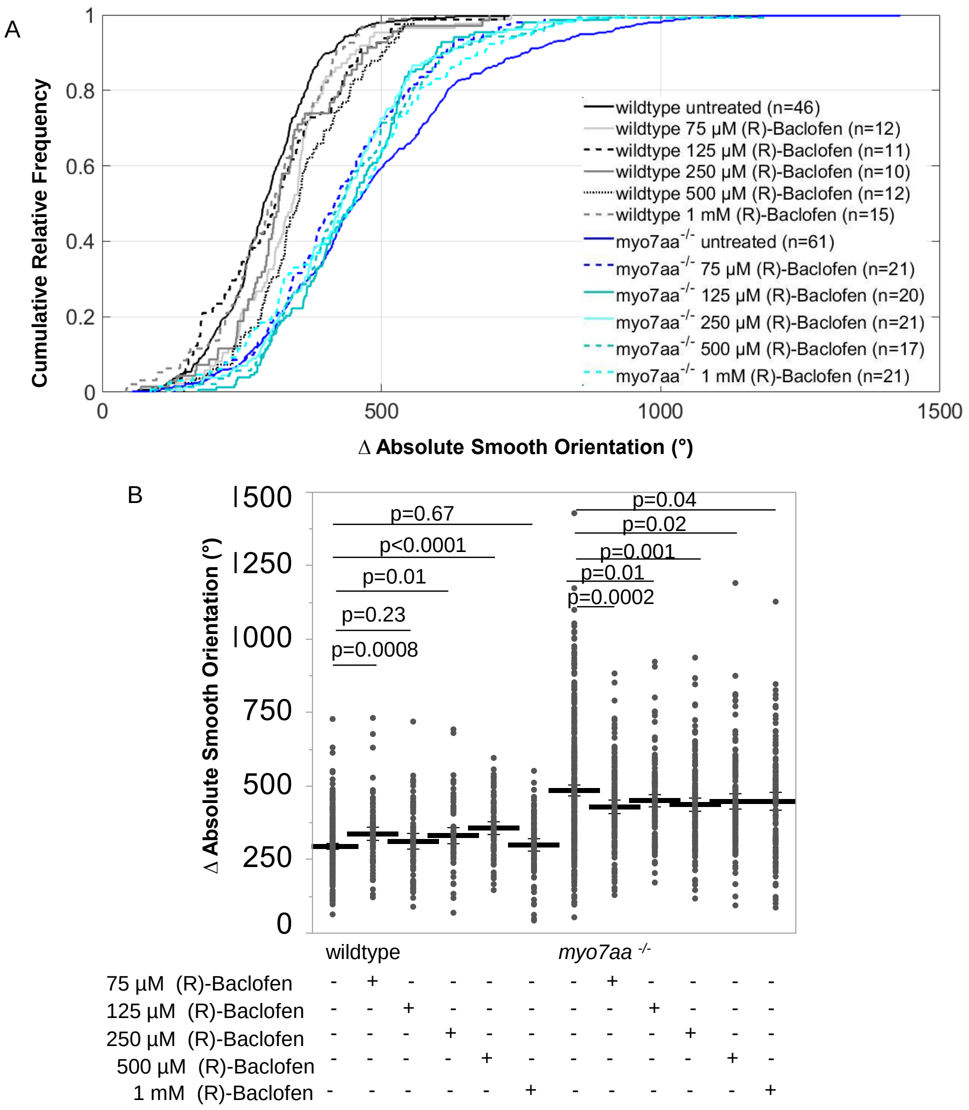

Fig. S6 Different doses of (R)-Baclofen have different effects on wildtype and *myo7aa*$^{-/-}$ mutant swimming behavior. (A, B) Incubation with 125 µM and 1 mM (R)-Baclofen had no adverse effects on wildtype swimming behavior; however incubation with 75 µM, 250 µM and 500 µM increased the absolute smooth orientation (turning angle as a function of time) of wildtype larvae. Incubation with all 5 doses of (R)-Baclofen did decrease the absolute smooth orientations of *myo7aa*$^{-/-}$ mutants compared to those untreated. Individual turning angles from populations of wildtype and *myo7aa*$^{-/-}$ larvae treated and untreated were used to construct the lines (t-test). The black bold line represents the mean of the data set and error bars are 95% confidence intervals. These experiments were replicated twice for each dose for each genotype.

Disease Models & Mechanisms • Supplementary information

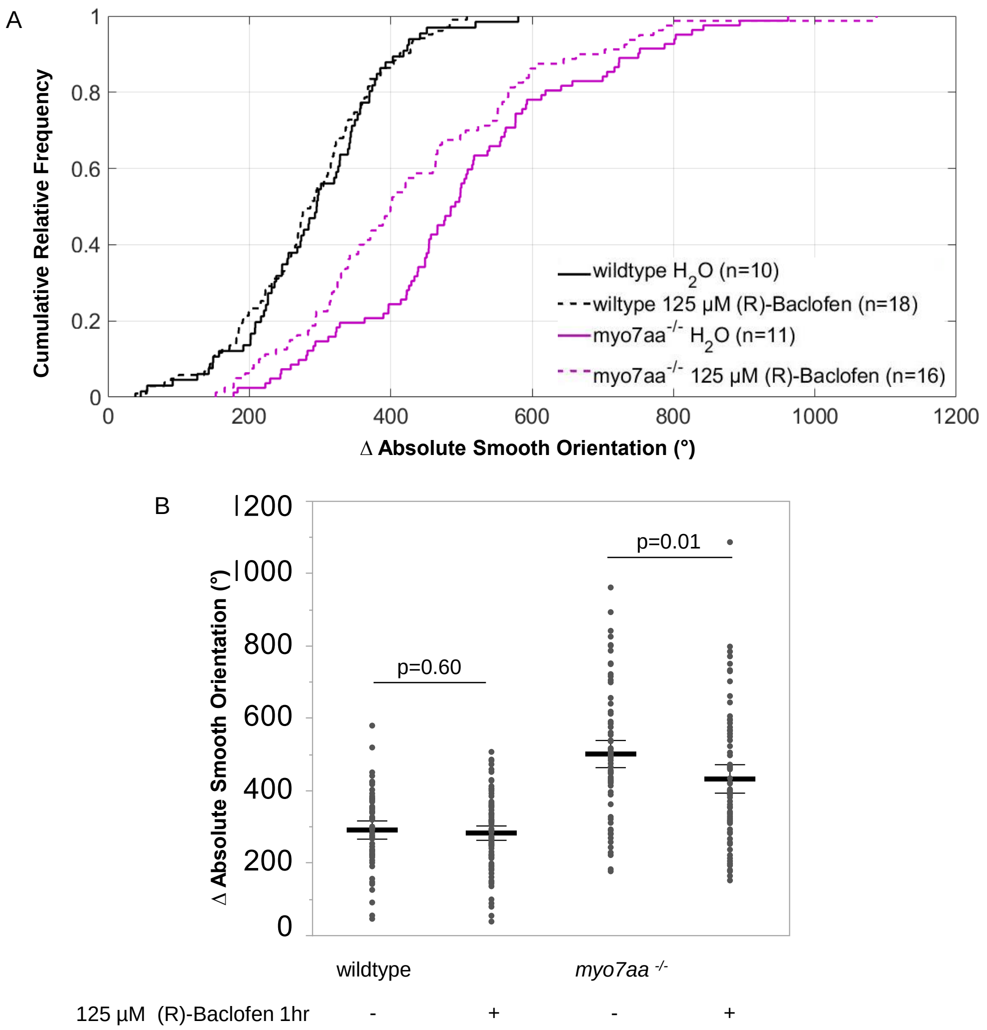

Fig. S7 1 hour incubation with (R)-Baclofen is sufficient to affect swimming behavior of *myo7aa*$^{-/-}$ mutants. In order to determine if a shorter incubation period would provide similar responses to an overnight incubation the absolute smooth orientation was quantified. (A, B) Incubation with 125 µM (R)-Baclofen for 1 hour had no adverse effects on wildtype swimming behavior and interestingly did decrease the absolute smooth orientation of the *myo7aa*$^{-/-}$ mutants, indicating that 1 hour incubation is sufficient for this drug. Individual turning angles from populations of wildtype and *myo7aa*$^{-/-}$ larvae treated and untreated were used to construct the lines (t-test). The black bold line represents the mean of the data set and error bars are 95% confidence intervals. These experiments were replicated twice for each dose for each genotype.

Disease Models & Mechanisms • Supplementary information

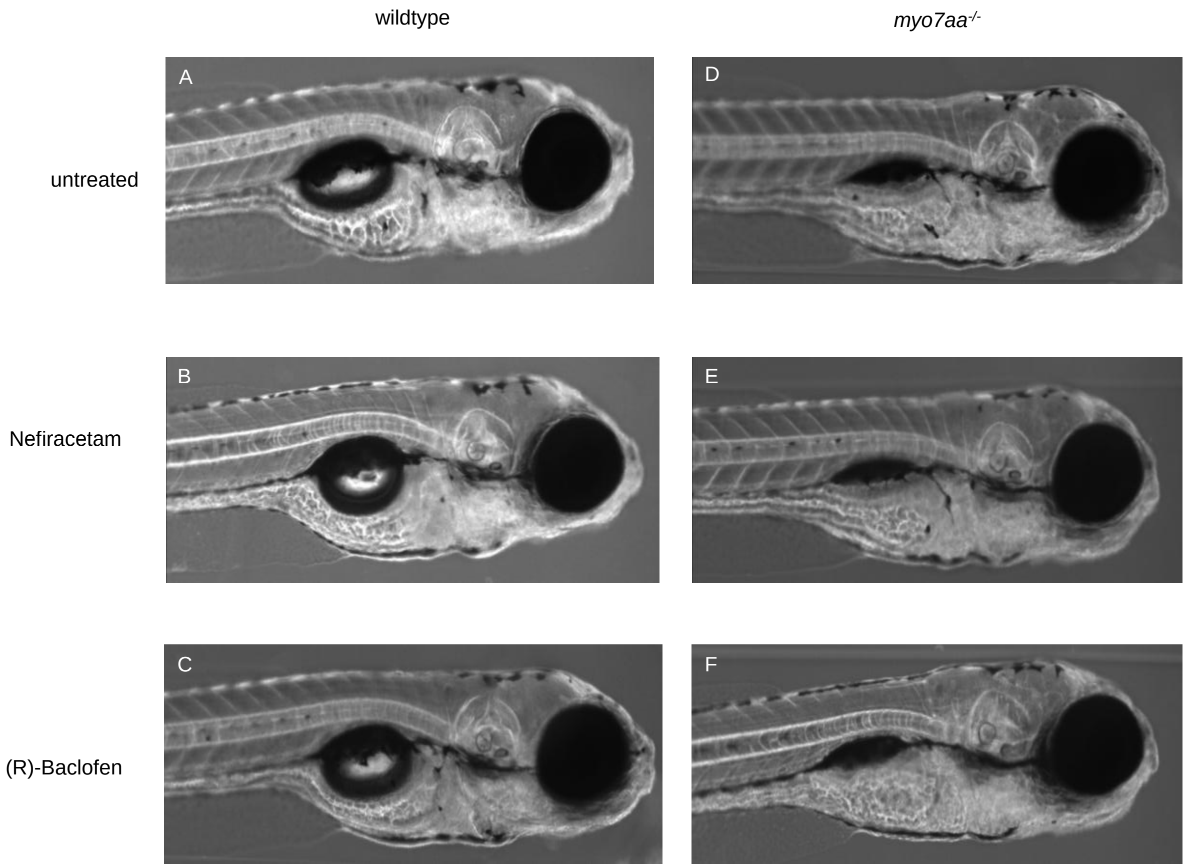

Fig. S8 250 µM Nefiracetam and 125 µM (R)-Baclofen have no effect on inflation of swim bladder on both wildtype and *myo7aa*$^{-/-}$ larvae. (A, B, C) At 5 dpf wildtype fish swim bladder inflation is unaffected upon incubation with either compound. (D, E, F) At 5 dpf *myo7aa*$^{-/-}$ mutant swim bladder inflation is unaffected upon incubation with either 250 µM Nefiracetam or 125 µM (R)-Baclofen. These experiments were replicated twice for each dose for each genotype.

Disease Models & Mechanisms • Supplementary information
